# Spreading of Tau Protein Does Not Depend on Aggregation Propensity

**DOI:** 10.1007/s12031-023-02143-w

**Published:** 2023-08-22

**Authors:** Sara Rodrigues, Marta Anglada-Huguet, Katja Hochgräfe, Senthilvelrajan Kaniyappan, Susanne Wegmann, Eva-Maria Mandelkow

**Affiliations:** 1 DZNE, German Ctr. for Neurodegenerative Diseases, Venusberg-Campus 1/99, 53127 Bonn, Germany; 2 CAESAR Research Center, Ludwig-Erhard-Allee 2, 53175 Bonn, Germany; 3 Department of Neurodegenerative Diseases and Gerontopsychiatry, University of Bonn Medical School, Bonn, Germany; 4 DZNE, German Center for Neurodegenerative Diseases, Chariteplatz 1, 10117 Berlin, Germany

**Keywords:** Alzheimer disease, Tau pathology, Tau spreading, Aggregation propensity, Neuroinflammation

## Abstract

The stereotypical progression of Tau pathology during Alzheimer disease has been attributed to trans-neuronal spreading of misfolded Tau proteins, followed by prion-like templated aggregation of Tau. The nature of Tau and the cellular mechanisms of Tau spreading are still under debate. We hypothesized that Tau’s propensity for aggregation would correlate with its ability to spread across synapses and propagate pathology. To study the progressive propagation of Tau proteins in brain regions relevant for Alzheimer disease, we used mice expressing near-physiological levels of full-length human Tau protein carrying pro-aggregant (TauΔK280, Tau^ΔK^) or anti-aggregant (TauΔK280-PP, Tau^ΔK−PP^) mutations in the entorhinal cortex (EC). To enhance Tau expression in the EC, we performed EC injections of adeno-associated virus (AAV) particles encoding Tau^ΔK^ or Tau^ΔK−PP^. The brains of injected and non-injected EC/Tau^ΔK^ and EC/Tau^ΔK−PP^ mice were studied by immunohistological and biochemical techniques to detect Tau propagation to dentate gyrus (DG) neurons and Tau-induced pathological changes. Pro- and anti-aggregant mice had comparable low transgene expression (~0.2 times endogenous mouse Tau). They accumulated human Tau at similar rates and only in expressing EC neurons, including their axonal projections of the perforant path and presynaptic terminals in the molecular layer of the DG. Pro-aggregant EC/Tau^ΔK^ mice showed misfolded Tau and synaptic protein alterations in EC neurons, not observed in anti-aggregant EC/Tau^ΔK−PP^ mice. Additional AAV-mediated expression of Tau^ΔK^ or Tau^ΔK−PP^ in EC/Tau^ΔK^ or EC/Tau^ΔK−PP^ mice, respectively, increased the human Tau expression to ~0.65 times endogenous mouse Tau, with comparable spreading of Tau^ΔK^ and Tau^ΔK−PP^ throughout the EC. There was a low level of transcellular propagation of Tau protein, without pathological phosphorylation or misfolding, as judged by diagnostic antibodies. Additionally, Tau^ΔK^ but not Tau^ΔK−PP^ expression induced hippocampal astrogliosis. Low levels of pro- or anti-aggregant full-length Tau show equivalent distributions in EC neurons, independent of their aggregation propensity. Increasing the expression via AAV induce local Tau misfolding in the EC neurons, synaptotoxicity, and astrogliosis and lead to a low level of detectable trans-neuronal spreading of Tau. This depends on its concentration in the EC, but, contrary to expectations, does not depend on Tau’s aggregation propensity/misfolding and does not lead to templated misfolding in recipient neurons.

**Supplementary Information:**

The online version contains supplementary material available at 10.1007/s12031-023-02143-w.

## Background

Alzheimer disease (AD) is a progressive age-related neurodegenerative disorder characterized by a gradual and progressive impairment in cognitive functions (Braak and Del Tredici 2016). Hyperphosphorylation and aggregation of the Tau protein and the formation of neurofibrillary tangles (NFTs) are hallmarks of AD (Grundke-Iqbal et al. 1986; Iqbal et al. 2011). Tau belongs to the family of microtubule-associated proteins (Dehmelt and Halpain 2005)and is expressed mainly in the axons of neurons (Götz et al. 2013), although it is also present—at lower levels — in astrocytes and oligodendrocytes (Lopresti et al. 1995; Gorath et al. 2001). Tau has a central role in microtubule (MT) stabilization and dynamics and in the regulation of axonal transport (Shahani and Brandt 2002). However, numerous other functions have been identified and suggested to play a role for Tau toxicity in neurodegenerative diseases (Chang et al. 2021).

The human Tau (hTau) gene (*MAPT*) is located on chromosome 17q21.1 and comprises sixteen exons resulting from alternative splicing (Neve et al. 1986; Goedert and Jakes 1990). By alternative mRNA splicing of exons 2, 3, and 10, six Tau isoforms are produced in the central nervous system (CNS), resulting in six different polypeptide chains with molecular weights between 35 and 70 kDa. The isoforms differ in the presence or absence of one or two short inserts in the amino-terminal half (0N, 1N, and 2N, respectively) and have either three or four semi-conserved repeats (~31 amino acid residues each) in the carboxy-terminal half (3R and 4R-Tau).

Under physiological conditions, Tau can be post-translationally modified at multiple sites, which affects the protein’s structure, function, and cellular processing (Buée et al. 2000; Guo et al. 2017). Phosphorylation has received most attention because it is believed that Tau pathology arises, at least in part, from the impaired ability of phosphorylated Tau to bind to MTs; cytosolic accumulation of phospho-Tau has been proposed to precede Tau aggregation, leading to neuronal degeneration in AD and tauopathies (Gong and Iqbal 2008).

Another characteristic of Tau pathology in AD is the progressive appearance of NFTs through the brain in a stereotypical anatomical pattern, which provides the basis for disease staging (Braak and Braak 1991): NFT pathology starts in the transentorhinal and entorhinal cortex (EC; Braak stages I and II) and progresses to the hippocampal formation (Braak stages III–IV). First clinical symptoms arise from neuronal impairment in this region (mild cognitive impairment). When NFTs progress further to neocortical areas (Braak stages V and VI), patients become severely demented, meeting the neuropathological criteria for the diagnosis of AD (Braak and Braak 1991; Clavaguera et al. 2014).

In a current model, the propagation of NFT pathology along neural connections is accounted to the ability of Tau proteins to transfer between cells (Vaquer-Alicea and Diamond 2019). This became first apparent in mice that express aggregating human mutant Tau in neurons of the EC and, later in life, show misfolded and hyperphosphorylated human Tau also in synaptically connected regions (de Calignon et al. 2012; Liu et al. 2012). By now, several in vitro and in vivo studies showed that Tau can be released and taken up by neighboring or distant cells via different mechanisms (Clavaguera et al. 2009; Frost et al. 2009; Guo and Lee 2011)whereby different isoforms and mutants seem to influence the spreading of Tau (Wegmann et al. 2019; Dujardin et al. 2018). Notably, all these studies used (in part high) overexpression of human Tau to induce and study the spread to downstream neurons, which likely influences not only the spreading rate but also the misfolding and aggregation of Tau in the brain. Hence, it is still not clear, which Tau species can be transferred from cell to cell and whether these species are responsible for the development of pathology at physiological Tau levels (Walsh and Selkoe 2016).

Other factors may be needed for Tau spreading as well, for example, it has been proposed that microglia may be involved in the spreading of Tau in the brain (Asai et al. 2015)and that neuroinflammation may play a role in the onset and propagation of Tau pathology (Gorlovoy et al. 2009; Maphis et al. 2015; Hansen et al. 2018; Ising et al. 2019; Simon et al. 2019; Vogels et al. 2019). Reactive microglia and astrocytes and pro-inflammatory molecules are typically observed around NFTs in the brains of AD patients and animal models of the disease (Cras et al. 1991; DiPatre and Gelman 1997; Sheffield et al. 2000; Endepols et al. 2022).

To unravel if pathological changes in Tau — phosphorylation, misfolding, and aggregation — are prerequisites for Tau spreading between neurons at physiological Tau levels, we generated mice expressing low physiological levels of human full-length mutant pro-aggregant (Tau^ΔK280^) and anti-aggregant (Tau^ΔK280−PP^) Tau restricted to the EC. The ΔK280 mutation induces a high aggregation propensity of Tau (Eckermann et al. 2007; Anglada-Huguet et al. 2023). Two Ile-Pro substitutions (I277P and I308P) in the repeat domain of Tau^ΔK280^ produce the anti-aggregant version Tau^ΔK280−PP^, in which the formation of aggregates is inhibited. Higher expression levels of Tau^ΔK280^ and Tau^ΔK280−PP^ in these mice were achieved through targeted adeno-associated virus (AAV) injections into the EC, which allowed us to compare Tau spreading at different expression levels. In these mice, we determined Tau phosphorylation and misfolding, often associated with pathological states of Tau, and compared tau propagation from the EC to synaptically neurons in the dentate gyrus (DG).

## Materials and Methods

### Animals

All animal experiments were carried out in accordance with the guidelines of the German Welfare Act and approved by the local authorities (Landesamt für Natur, Umwelt und Verbraucherschutz Nordrhein-Westfalen) under animal permission 84–02.04.2016-A278. Animals were housed in groups of 2–5 animals under standard conditions (23 °C, 40–50% humidity, ad libitum access to food and water) with a 12-h light/dark cycle (with light on from 6 a.m. to 6 p.m.).

Transgenic mice expressing mutant pro- and anti-aggregant hTau were used. The “pro-aggregant” tTA-EC/Tau^ΔK^ mouse line (EC/Tau^ΔK^) was generated by crossing the responder line of transgenic mice co-expressing the human full-length human Tau protein (hTau40; 2N4R; UniprotKB P10636-8) with the FTDP-17 mutation ΔK280 (deletion of lysine 280, termed “pro-aggregant” Tau^ΔK^, 441–1 = 440 residues) and the reporter firefly luciferase gene under the control of a bidirectional tetO-responsive CMV promoter, with the activator mouse line Neuropsin (Nop)-tTA, which expresses the tetracycline-controlled transactivator (tTa) exclusively in the entorhinal cortex (EC) layer II, as described before (de Calignon et al. 2012; Liu et al. 2012; Eckermann et al. 2007; Yasuda and Mayford 2006; Dennissen et al. 2016). This yielded a regional neuron-specific expression of mutant hTau and luciferase confined to the medial EC (MEC). In parallel, the “anti-aggregant” tTA-EC/Tau^ΔK−PP^ mouse line (EC/Tau^ΔK−2P^ for short) was generated with the same constructs as the tTA-EC/Tau^ΔK^ mouse, but with two additional Ile-Pro substitutions (I277P and I308P) in the hexapeptide motifs of the repeat domain of the Tau protein, which serve as β-sheet breakers (termed Tau^ΔK−PP^) (Eckermann et al. 2007; von Bergen et al. 2000). Wild-type (WT) C57BL-6 J mice and mice lacking Tau protein (Tau-KO, B6.129X1-Mapttm1Hnd/J (Mapt0/0)) (Dawson et al. 2001) were also used in the experiments. In addition, the level of Tau^ΔK^ or Tau^ΔK−2P^ in the EC of transgenic or control mice was further increased by injection of AAV encoding these proteins (see below). We analyzed gender-mixed animals. Transgenic mouse lines tTA-EC/Tau^ΔK^ and tTA-EC/Tau^ΔK−PP^ were identified by PCR using the following primer pairs: hTau transgene (JB309/pBI5-BN): forward 5′-GAC CTT CCG CGA GAA CGC CAA A-3′; reverse 5′-AAG AAC AAT CAA GGG TCC CCA-3′; neuropsin promoter (Nop-for/Nop-rev): forward 5′-ACC GAG AAG CAG TAC GAG A-3′; reverse 5′-ACT CGC ACT TCA GCT GCT T-3′.

### Luciferase Bioluminescence

Whole brain ex vivo slices were prepared from 6 months old pro- and anti-aggregant mice as previously described (Humpel et al. 2018). Briefly, mice were anesthetized (Isoflurane, Piramal Critical Care, Germany) and sacrificed by cervical dislocation, and the head was removed using a pair of scissors. After dissecting the brain, 500-μm-thick horizontal brain slices were prepared using a vibratome (Leica VT1200, Germany). Slices were immediately transferred onto membrane inserts in 6-well plates and kept alive in slice culture media containing 0.15 mg/mL D-luciferin, at 37 °C, 5% CO_2_incubator (three whole brain sections per membrane). A subsequent BLI scan (IVIS Lumina II system; Caliper Life Science, USA) exhibited the transgene expression pattern (Hochgrafe and Mandelkow 2013).

In vivo BLI was performed using the Ivis Lumina II system according to a standardized protocol (Hochgräfe and Mandelkow 2013). Ten minutes before the imaging session the mice received an intraperitoneal (i.p.) injection of 150 mg/kg of D-luciferin (Caliper Life Science, USA) dissolved in sterile PBS. The heads of the mice were shaved to avoid optical attenuation of emitted photons. Mice were anesthetized with 2% Isoflurane (Piramal Critical Care, Germany) in a constant O_2_ flow maintained throughout the whole imaging session. Mice were placed into the heated, light-tight imaging chamber, and a sequence of 6 images, taken in intervals of 2 min starting 10 min after D-luciferin injection, was recorded using a highly sensitive charged coupled device camera. The analysis of the recorded images was accomplished using Living Image 4.0 software (Caliper Life Science, USA). The BLI emission was normalized, and the surface radiance was displayed in photons per second per square centimeter per steradian (photons/s/cm^2^/sr). For quantification of BLI signals, a region of interest (ROI) was defined to convert surface radiance (photons/s/cm^2^/sr) into total flux of the bioluminescent source (photons/s) (Hochgrafe and Mandelkow 2013).

### Adeno-associated Virus

Three adeno-associated virus (AAV) constructs were used to express Tau in mouse brains, following previous procedures (Wegmann et al. 2019):

1. 1AAV encoding eGFP, the translation interrupting 2a peptide, and pro-aggregant full length mutant human Tau^ΔK^ (AAV2/8-CBA-eGFP-2a-hTau40^ΔK280^ (named AAV-Tau^ΔK^); titer = 9.71 × 10^11^GC/ml);
2. AAV encoding eGFP, the translation interrupting 2a peptide, and anti-aggregant full length mutant human Tau^ΔK−PP^ (AAV2/8-CBA-eGFP-2a-hTau40^ΔK280−PP^ (named AAV-Tau^ΔK−PP^); titer = 1.65 × 10^12^GC/ml); and
3. AAV encoding eGFP (AAV2/8.CBA.eGFP-2a WPRE.Bgh (named AAV-GFP)), titer 1.37 × 10^13^ GC/ml). All AAV constructs were produced at Gene Transfer Vector Core (GTVC), Schepens Eye Research Institute and Massachusetts Eye and Ear Infirmary, Harvard Medical School. The plasmid maps are presented in Suppl. Fig. S1.

### Stereotaxic Injections

Intracerebral injections of AAV into the brain of anesthetized (Isoflurane, Piramal Critical Care, Germany) were performed on 5-month-old tTA-EC/Tau^ΔK^, tTA-EC/Tau^ΔK−PP^, WT and Tau-KO mice following published procedures (Spires-Jones et al. 2011; Wegmann et al. 2017). For unilateral expression, AAV encoding mutant human Tau or eGFP was injected in the right EC using classic stereotaxic procedures at the following coordinates: AP − 4.7 mm, ML + 3.6 mm (from Bregma), DV − 3.0 (from dura mater/brain surface)). The contralateral (left) hemisphere was injected with PBS at the same brain coordinates as control. The standard injection procedure consisted in delivering 2 μL of AAV or PBS using a 10 μL glass syringe with a fixed needle (WPI, Germany). After reaching the injection coordinates, the needle was left in place for 2 min to allow the tissues to adapt. After injection at a rate of 0.2 nL/min, the needle was left in place for an additional 5 min to prevent backflow of the injected solution.

AAV-Tau^ΔK^ was injected into tTA-EC/Tau^ΔK^ in order to boost levels of Tau^ΔK^, and as controls into WT and Tau-KO mice. Similarly, AAV-Tau^ΔK−PP^ was injected into tTA-EC/Tau^ΔK−PP^, WT, and Tau-KO mice. As a control, a group of WT mice was injected with AAV-GFP. Mice were sacrificed by cervical dislocation at 3, 6, 12, and 18 months p.i. (ages ~8, 11, 17, 23 mo), and the brains were collected for analysis.

### Histological Analysis

Mice were anesthetized (Isoflurane, Piramal Critical Care, Germany) and sacrificed by cervical dislocation. The brains were removed and fixed in histofix (Carl Roth, Germany; 4% PFA, pH 7.4 for 24 h) and dehydrated with ethanol and chloroform, followed by embedding in paraffin. Horizontal 5-μm-thick paraffin brain sections were cut on a microtome (Microtome Slide 2003, Pfm Medical AG, Germany) and mounted onto superfrost plus adhesion microscope slides (Thermo Fisher Scientific, Germany). Sections were deparaffinized at 60 °C for 10 min and rehydrated by incubation with decreasing xylene and ethanol solutions finishing in ddH_2_O. Antigen retrieval with citrate buffer at 80 °C for 30 min was performed, and sections were permeabilized with TBS-Triton X-100 0.1% 3 × 10 min. Non-specific binding sites were blocked with 5% normal horse serum for 60 min at room temperature (RT), and slices were incubated with primary antibody in 1% blocking serum overnight at 4 °C. The following antibodies were used: HT7 (human Tau specific, 1:1000, Thermo Fisher Scientific), 12E8 (Tau phosphorylated at pS262/pS356, 1:2000, ELAN Pharmaceuticals), Iba1 (microglia, 1:1000, Wako), GFAP (astrocytes, 1:2000, Sigma-Aldrich). On the second day, slides were washed 3 × 10 min with TBS 0.1% Triton X-100 and incubated in biotinylated secondary antibody for 60 min at RT. Slides were washed 3 × 10 min in TBS 0.1% Triton X-100 and incubated with avidin–biotin-peroxidase complex (ABC) solution (Vectastain ABC kits, Vector Laboratories Inc., USA) in 10% blocking serum in TBS 0.1% Triton X-100 for 60 min at RT. Afterwards, sections were washed 3 × 10 min in TBS 0.1% Triton X-100 and incubated in DAB solution (30 µL of DAB chromogen (reagent B) to 1 mL DAB substrate buffer (reagent A)) until staining was optimal as determined by light microscopic examination. The reaction was stopped in tap water, and sections were dehydrated by incubation with increasing ethanol and xylene solutions and coverslips mounted with Roti^®^-Histokitt (Carl Roth, Germany).

### Immunofluorescence

Mice were anesthetized (Isoflurane, Piramal Critical Care, Germany) and sacrificed by cervical dislocation. The brains were drop-fixed in 4% histofix (Roth; 4% PFA, pH 7.4) for 3 days, cryoprotected in 30% sucrose in PBS with 0.02% sodium azide, frozen embedded in Shandon™ Cryomatrix™ embedding resin (Thermo Fisher Scientific, Germany), cut into 40-µm-thick horizontal sections, placed on 96 well-plates (filled with 0.02% sodium azide in 1X PBS solution) as free-floating sections, and stored at 4 °C. Sections were washed with 1 × PBS and incubated 2 × 15 min in 50 mM NH_4_Cl. Sections were permeabilized with 1 × PBS 0.5% Triton X-100 (2 × 10 min) and blocked in 1 × PBS + 0.2% BSA + 0.5% Triton X-100 + 0.5% FBS for 1.5 h, followed by incubation with primary antibody in blocking buffer for 3 overnights at 4 °C with gentle agitation. The following antibodies were used: Tau Y9 (polyclonal, human Tau, 1:100, Enzo Life Sciences), MC1 (pathological conformation of Tau, 1:50, kind gift from Dr. P. Davies), PHF-1 (phosphorylated Tau, 1:100, kind gift from Dr. P. Davies), Iba1 (microglia, 1:100, WAKO), and GFAP (astrocytes, 1:250, Sigma-Aldrich). Afterwards, sections were washed 3 × 10 min in 1 × PBS and incubated with secondary antibodies overnight at 4 °C with gentle agitation. The following secondary antibodies (Dianova) were used: donkey Cy3 α-mouse (1:500), goat Cy3 α-rabbit (1:500), donkey Alexa 647 α-mouse (1:500), and Donkey Alexa 647 α-rabbit (1:500). Sections were washed 3 × 10 min in 1 × PBS, incubated 5 min with Hoechst solution (Thermo Fisher Scientific, Germany) 1:10 000 in 1 × PBS, washed 3 × 10 min in 1 × PBS, and mounted onto glass slides using Fluoromount-G mounting medium (Southern Biotech, Germany) and coverslipped.

### Brain Homogenization and Protein Quantification

After sacrificing the mice by cervical dislocation, the brains were collected immediately, and the following regions were dissected from both hemispheres (AAV-injected and PBS-injected controls, respectively) and stored at − 80 °C: EC, hippocampus, and cortex. Each of these was subdivided into 2 pieces, one for western blot and the other for sarkosyl extraction. Lysis buffer was added to each Eppendorf tube containing the dissected brain tissues (300 µL for the EC, 600 µL to the hippocampus and cortex), and samples were sonicated 5 s (amplitude 40%) followed by another sonication of 3 s. Samples were kept on ice for 30 min, centrifuged 20 s at 14,000 rpm (Eppendorf centrifuge 5415R, Germany), and the supernatant was collected. Protein concentration was estimated using 1 µL of the supernatant and a Bicinchoninic Acid Protein (BCA) assay kit (Sigma-Aldrich, Germany).

### Sarkosyl Extraction

A sarkosyl-insoluble Tau fraction was isolated from brain tissue as previously described (Greenberg and Davies 2006; Mocanu et al. 2008). Briefly, the brain tissue was weighed, homogenized in 3 × volume of cold Buffer H (10 mM Tris–HCl, 1 mM EGTA, 0.8 M NaCL, 10% sucrose, pH 7.4) and centrifuged at 26,000 rpm (Beckman CoulterTM Optima TM MAX-E) for 20 min at 4 °C. The supernatant was collected, and the resulting pellet was homogenized in buffer H and centrifuged at 26,000 rpm for 20 min at 4 °C. Both supernatants were combined, adjusted to 1% (w/v) N-lauroylsarcosine and incubated at 37 °C with shaking for 2 h. After centrifugation at 61,000 rpm for 35 min at 20ºC, the supernatant was collected (sarkosyl-soluble fraction) and the pellet was resuspended in 500 µL of 1 × TBS and centrifuged again at 61,000 rpm for 35 min at 20 °C. The supernatant was then removed, and the pellet resuspended in 0.5 µL 1 × TBS for each mg of original sample plus the same amount of 2 × sample buffer and samples stored for SDS gel. Western blotting was used to analyze the supernatant (sarkosyl-soluble fraction) and the pellet (sarkosyl-insoluble fraction).

### Western Blotting

Homogenized brain tissues (from EC, hippocampus and cortex, respectively) plus sarkosyl-soluble and sarkosyl-insoluble fractions were resolved in 10% SDS-PAGE gels, followed by semi-dry transfer to PVDF membranes (Carl Roth, Germany). Primary antibody incubation was performed overnight at 4 °C in TBS-T (Tris-buffered saline, 0.1% Tween 20) plus 5% nonfat dry milk. The following primary antibodies were used: 12E8 (1:2000, ELAN Pharmaceuticals), PHF-1 (1:1000, kind gift from Dr. P. Davies), K9JA (1:20 000, DAKO A0024), CD11b (1:1000, Abcam), synaptophysin (1:5000, Sigma-Aldrich), and GFAP (1:2000, Sigma-Aldrich). After washing 3 × in TBS-T, the membranes were incubated with secondary antibodies for 2 h at RT (anti-mouse 1:2000, or anti-rabbit 1:2000, DAKO). Antibody affinity was detected by chemiluminescence with Amersham ECL Prime Western Blotting Detection Reagent (GE Healthcare, Germany). Protein bands were visualized using Image Quant LAS 4000 mini (GE Healthcare Life Sciences, Germany), and band intensities were analyzed using Image Studio Lite 5.2 software (LI-COR Biosciences). Actin (1:10 000, Sigma-Aldrich, Germany) was used as loading control.

### Statistical Analysis

For western blotting, the mean density and area of each band were measured using at least three independent experiments in Image Studio Lite 5.2 software (LI-COR Biosciences, Germany). The statistical analysis was completed using Graph Pad (Prism) version 7.05 software. All values are given as mean ± SEM. To compare the experimental groups (3–4 animals/group), a one-way or two-way ANOVA was performed, with uncorrected Fisher’s LSD or Tukey’s post hoc test for multiple comparisons to evaluate statistical significance. Differences were considered statistically significant when *p* < 0.05.

## Results

### Entorhinal Restriction of Pro- and Anti-aggregant Human Tau in EC/Tau^ΔK^ and EC/Tau^ΔK−PP^ Mice 

**Fig. 1:**
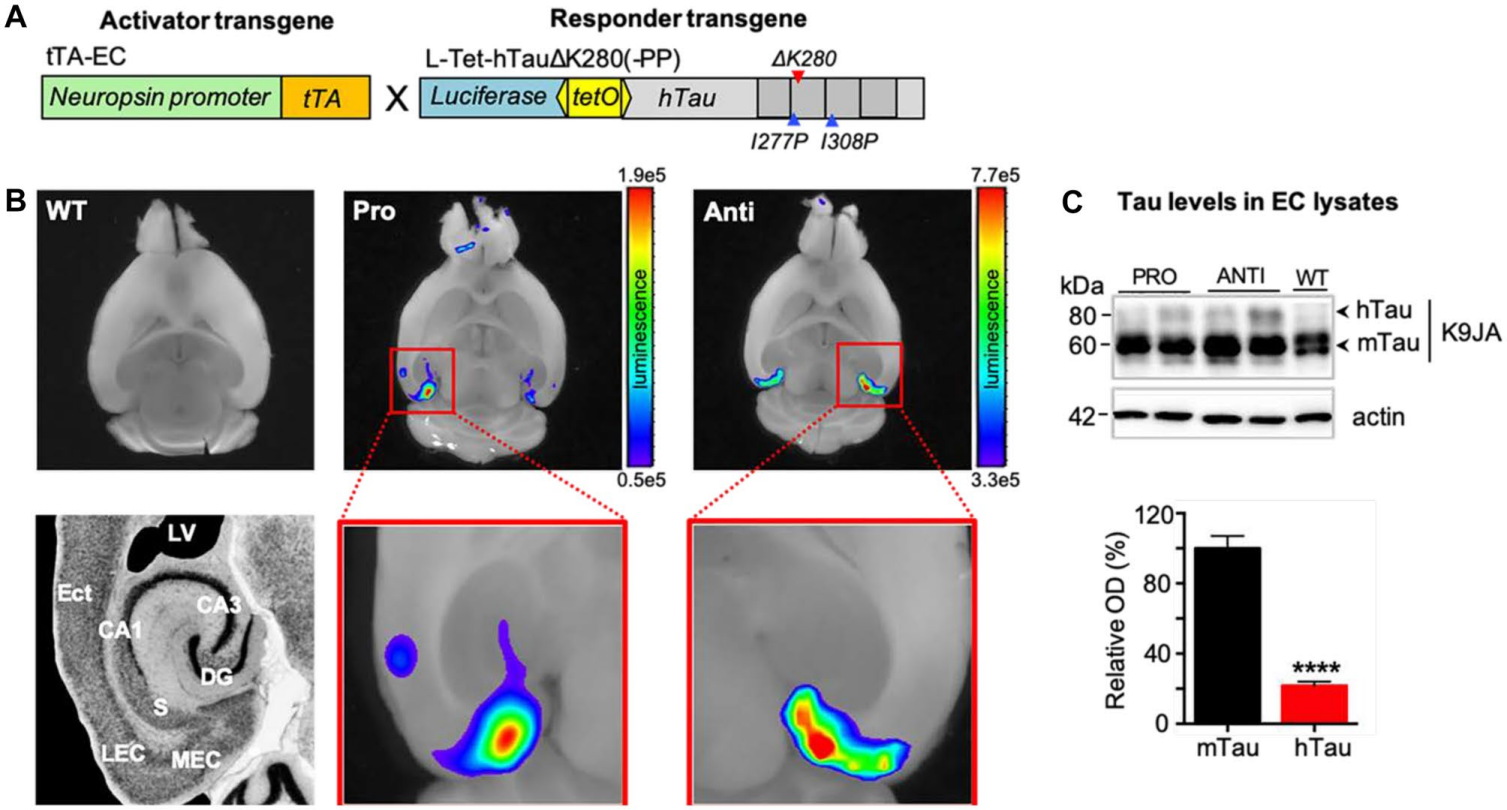
Expression of human mutant Tau in the entorhinal cortex of EC/Tau^K^ and EC/Tau^ΔK−PP^ mice. **A** Transgenic EC/Tau^ΔK^ and EC/Tau^ΔK−PP^ mice express the activator, tTA (orange), under the neuropsin promoter (green) and the responder genes, hTauΔK280 or hTauΔK280-PP (grey), and luciferase (blue) under a bi-directional *tetO* promoter (yellow). **B** Analysis of transgene expression in fresh ex vivo horizontal brain slices of 6-month-old animals shows the spatial restriction of hTau transgene expression in the EC from pro-aggregant (Pro) EC/Tau^ΔK^ and anti-aggregant (Anti) EC/Tau^ΔK−PP^ mice but not from wild-type (WT) mice. Slices were kept alive in slice culture media containing 0.15 mg/ml D-luciferin. For the quantification of BLI signals, surface radiance was measured as total flux of the bioluminescent source in photons per second. **C** Top: Western blot analysis of Tau in EC lysates using K9JA, an antibody for total tau, shows that 12-month-old pro- and anti-aggregant mice express low levels of hTau compared to endogenous mouse Tau (mTau). Bottom: Quantification (optical density; OD) of immunoblots reveals that hTau levels, across EC/Tau^ΔK^ and EC/Tau^ΔK−PP^ mice, correspond to ~20% of endogenous mTau (unpaired *t*-test; *p* < 0.0001). Data shown as mean ± SEM

To study the influence of Tau aggregation on its spread (cell-to-cell transfer) in the brain, we generated two mouse models with near-physiological expression levels of mutant pro-aggregant (TauΔK280, EC/Tau^ΔK^ line) and anti-aggregant (TauΔK280-PP, EC/Tau^ΔK−PP^ line) human full-length Tau (2N4R isoform) under the neuropsin promoter (Fig. 1A). The expression of transgenic human Tau in the brains of these mice is regulated by a bidirectional *tetO*promoter and can be monitored by luciferase bioluminescence imaging (BLI) (Eckermann et al. 2007). Accordingly, fresh ex vivo brain slices from 6-month-old pro-aggregant EC/Tau^ΔK^ and anti-aggregant EC/Tau^ΔK−PP^ mice presented strong luciferase signals, demonstrating human Tau (hTau) expression in the entorhinal region (Fig. 1B). Slices from WT mice did not show luciferase activity, hence hTau expression. Western blot analysis of total Tau in EC lysates from 12-month-old EC/Tau^ΔK^ and EC/Tau^ΔK−PP^ mice revealed low expression of hTau, which corresponded to ~20% of endogenous mouse Tau (mTau) (ratio hTau:mTau = 1:5) (Fig. 1C).

**Fig. 2:**
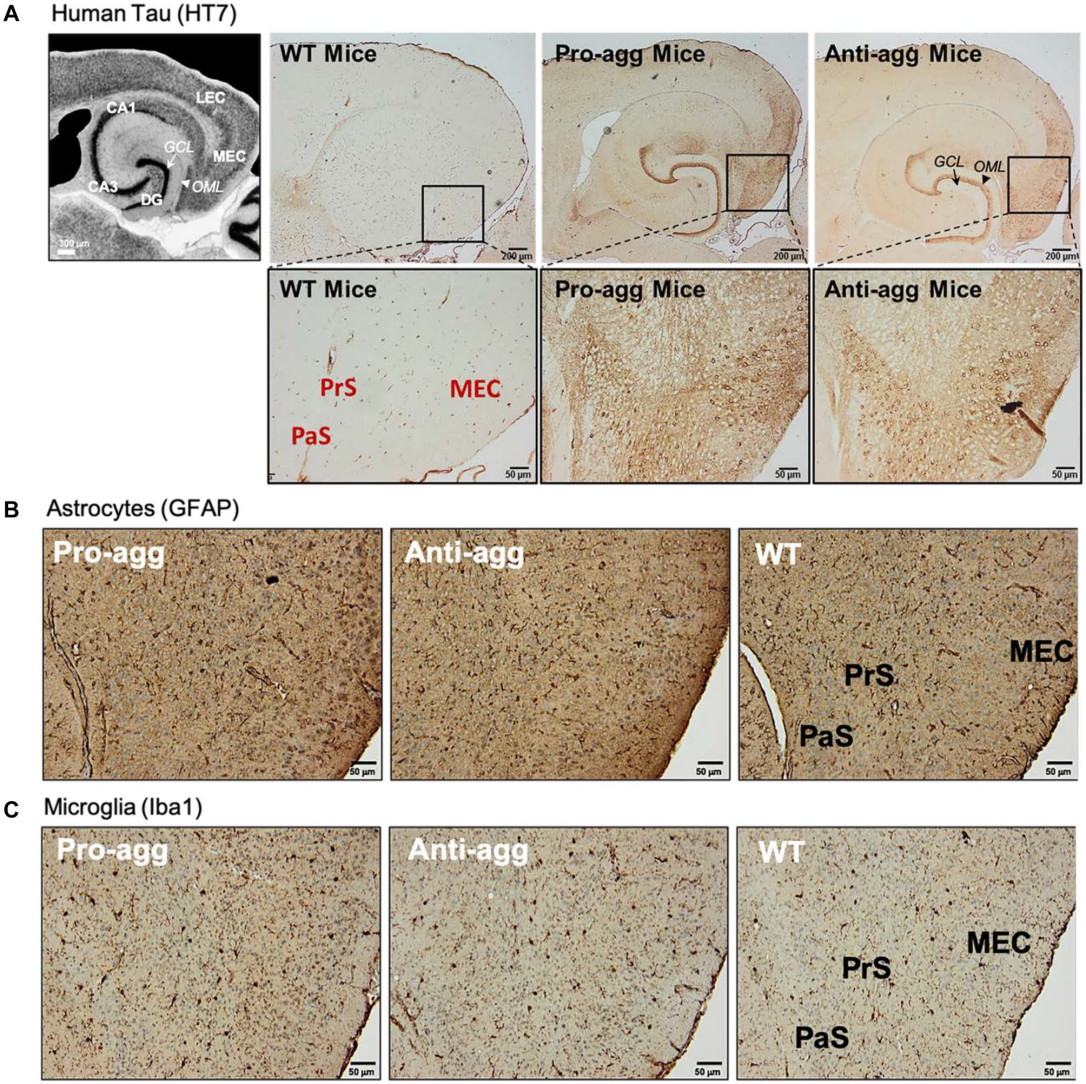
Restricted expression of mutant hTau and total Tau levels in the entorhinal cortex of pro- and anti-aggregant mice. **A** Human Tau expression in EC/Tau^ΔK^ (Pro-agg) and EC/Tau^ΔK−PP^ (Anti-agg) mice. Left, overview of hippocampal region (staining with DAPI, scale bar: 300 μm). Right: Immunolabeling of hTau (hTau-specific antibody HT7) in brain sections of 12-month-old mice shows the expression of hTau in neurons of the medial EC (MEC) and subiculum (PrS = presubiculum, PaS = parasubiculum), including their cell bodies, projections in the perforant pathway, and axon termini in the outer molecular layer (OML) adjacent to the granule cell (GCL) layer of the DG. Scale bar: 200 μm (upper figures); 50 μm (lower figures). **B** Astrocytes, detected by GFAP immunolabeling, show a similar distribution in the EC and hippocampus of EC/Tau^ΔK^ (Pro-agg) and EC/Tau^ΔK−PP^ (Anti-agg) mice, and WT mice at 24 months of age. Scale bar: 50 μm. **C** Microglia, detected by Iba1 immunolabeling, show a similar distribution in the EC and hippocampus of EC/Tau^ΔK^ (Pro-agg) and EC/Tau^ΔK−PP^ (Anti-agg) mice, and WT mice at 24 months of age. Scale bar: 50 μm

Immunolabeling of hTau using the human Tau-specific antibody HT7 in fixed brain sections of 12-month-old EC/Tau^ΔK^ and EC/Tau^ΔK−PP^ mice identified hTau in cell bodies and axons of EC neurons and in their axon terminals in the middle molecular layer of the dentate gyrus (DG) (Fig. 2A). No hTau was detected in WT mice. Immunohistological analysis of astrocytes (Fig. 2B) and microglia (Fig. 2C) showed no obvious increase in the number or changes in the distribution of glia cells between transgenic and WT mice, indicating no overt gliosis upon low expression of pro- or anti-aggregant hTau in EC/Tau^ΔK^ and EC/Tau^ΔK−PP^ mice, even after 24 months.

Notably, in contrast to previous studies using a similar mouse model (de Calignon et al. 2012), no hTau was detected in the granule cell layer of the DG and the pyramidal cell layers of CA1 or CA3, which are directly synaptically connected to the EC. Similarly, also in 24-month-old in EC/Tau^ΔK^ and EC/Tau^ΔK−PP^ mice, hTau was only visible in EC neurons and axons traversing the perforant pathway (Suppl. Fig. S1). The absence of hTau in neurons of the DG indicated that no major trans-synaptic propagation of hTau had occurred, given the low expression level of EC Tau in the mice used here.

### Enhanced Tau Phosphorylation and Misfolding in EC/Tau^ΔK^ Mice

**Fig. 3:**
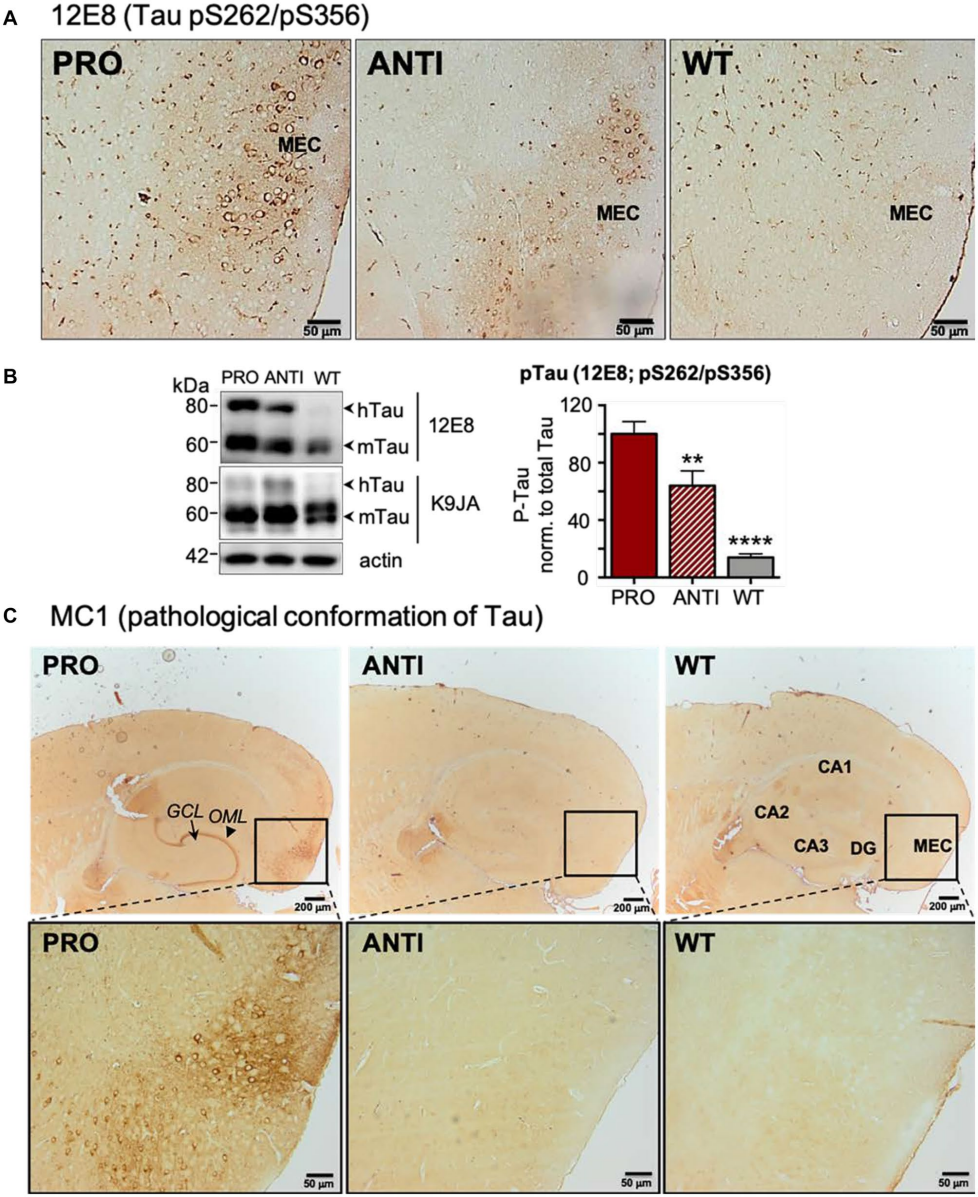
Tau phosphorylation and pathological conformation are prominent in EC/Tau^ΔK^ pro-aggregant mice. **A** Neurons in the MEC show accumulation of Tau phosphorylation at Ser262/Ser356, detected with the 12E8 antibody, in the cell bodies (round shapes) at 6 months of age. The EC of EC/Tau^ΔK^ (PRO) shows substantially more 12E8 + cells compared to EC/Tau^ΔK−PP^ (ANTI) mice. No 12E8 + cells are found in WT mice. Scale bar: 50 μm. **B** Left: Western blot analysis of EC lysates from 6-month-old mice shows that the levels of phospho-Tau (12E8) compared to total Tau (K9JA) are significantly higher in EC/Tau^ΔK^ (PRO) mice (lane 1) than in EC/Tau^ΔK−PP^ (ANTI; *p* = 0.0021, lane 2) or WT mice (*p* < 0.0001, lane 3). Interestingly, the effect of Tau^ΔK^ expression was observed for both transgenic human (hTau) and endogenous mouse (mTau). Right: Quantification. One-way ANOVA; post hoc: uncorrected Fisher’s LSD test. Data shown as mean ± SEM. **C** Pathological conformation of Tau in 6-month-old mice, detected with MC1 antibody, was present only in hTau expressing neurons of EC/Tau^Δ^.^K^ (PRO) mice, in the EC and along the perforant path up to the axon terminals where it accumulated in the OML of the DG. Scale bar: 200 μm (upper images); 50 μm (lower images)

Next, we analyzed the effect of pro- and anti-aggregant Tau expression on Tau phosphorylation and pathological conformation. At 6 months of age, Tau phosphorylation at Ser262/Ser356 (detected with 12E8 antibody) was observed in EC/Tau^ΔK^ and, less pronounced, in EC/Tau^ΔK−PP^ mice (Fig. 3A, B), indicating some shift in the kinase/phosphatase balance. Tau with a pathological conformation (as judged by antibody MC1 (Jicha et al. 1997) was detected in EC/Tau^ΔK^ mice in the soma, axonal projections, and axon terminals, but was absent in EC/Tau^ΔK−PP^ or WT mice (Fig. 3C).

Notably, no misfolded Tau was found in cells of the DG and CA1/CA3, synaptically connected to EC neurons, even up to 24 months of age (Suppl. Fig. S2).

These results show that both pro- and anti-aggregant mutant Tau expressed at low amounts in EC neurons is transported to the axon terminals but does not spread to downstream neurons (during the observed 24 months). This observation is expected for an axonal protein transported by slow axonal transport (Mercken et al. 1995). In particular, the induced pre-tangle conformation of Tau revealed by antibody MC1, an early hallmark of Tau pathology, was observed selectively in EC/Tau^ΔK^ neurons. By comparison, mouse models with ~ threefold overexpression of human FTD-mutant Tau^P301L^in the EC showed propagation to the DG after 15–18 months (de Calignon et al. 2012; Liu et al. 2012). Thus, considering the low levels of hTau expression in EC/Tau^ΔK^ and EC/Tau^ΔK−PP^ mice, we hypothesize that increasing the hTau expression levels in the EC of these mice would accelerate Tau propagation and pathology.

### Increased AAV-Mediated Tau^ΔK^ and Tau^ΔK−PP^ in the EC of in EC/Tau^ΔK^ and EC/Tau^ΔK−PP^ Mice

**Fig. 4:**
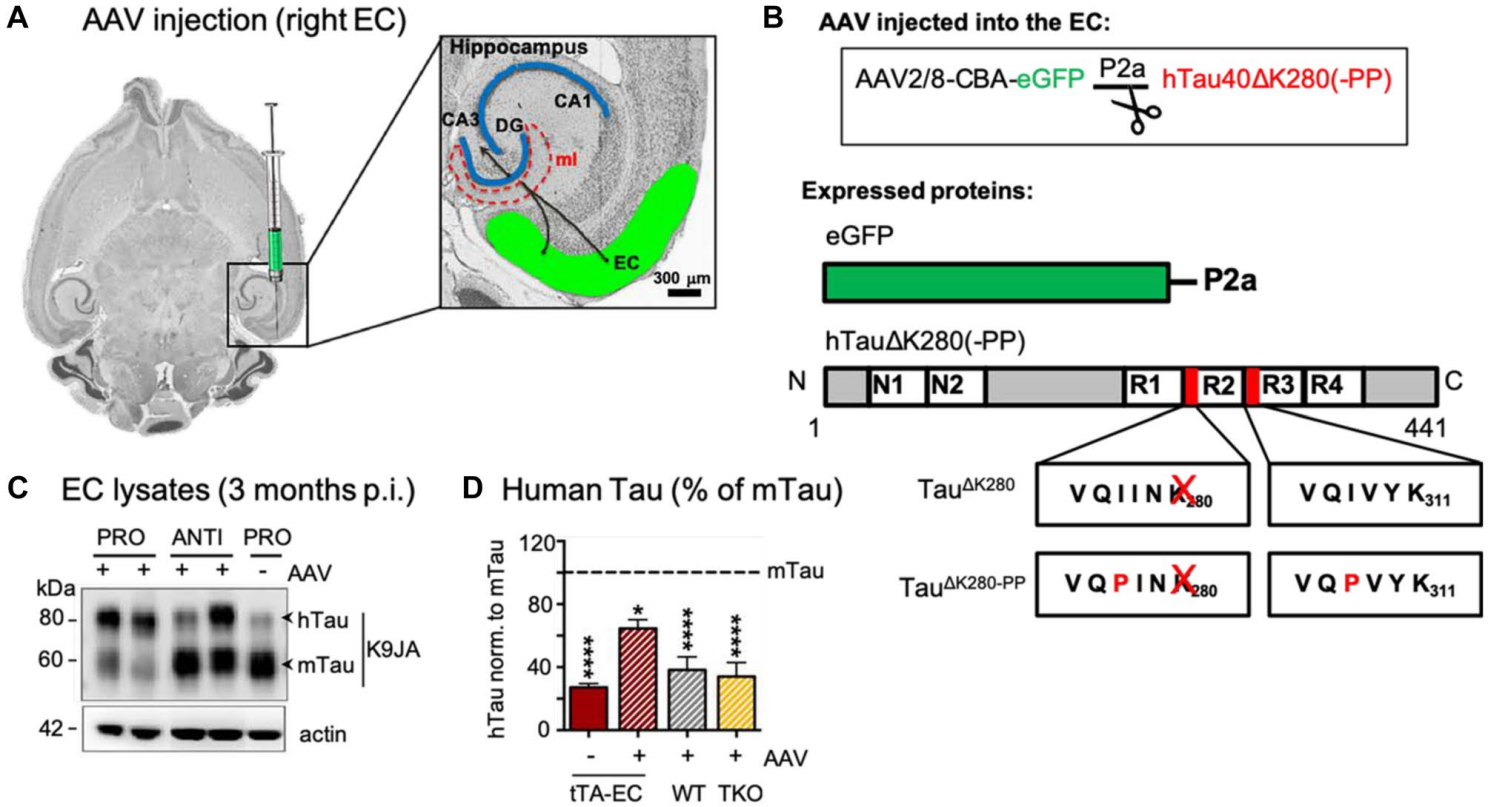
AAV eGFP-P2A-hTauK280(-PP) injections increase hTau levels in the EC of EC/Tau^ΔK^ and EC/Tau^ΔK−PP^ mice. **A** Schematic drawing of unilateral AAV injections into right EC, from where the axons of EC neurons project towards the perforant path and synaptically connect to the dendrites of neurons emanating from the DG cell bodies of the granule layer to the molecular layer (ml). **B** Top: AAV constructs used are coding for one mRNA containing the DNA for eGFP as transfection marker, and hTau^ΔK280^ (AAV/Tau^ΔK^) or hTau^ΔK280−PP^ (AAV/Tau^ΔK−PP^). Below: During translation, the short 2a peptide (P2a) causes ribosome stuttering and thereby the production of eGFP (green) and hTau (gray) as two separate proteins. The diagram represents the Tau isoform 2N4R (441 residues). The two N-terminal inserts (N1, N2) and the four repeats (R1–R4) are indicated. The two hexapeptides with high propensity for β-structure at the beginning of R2 and R3 are indicated, as well as the positions of the pro-and anti-aggregant mutations. **C** At 3 months post-injection (= 8 months of age), WB analysis of K9JA (reporting on total Tau), showed that both EC/Tau^ΔK^ injected with AAV/Tau^ΔK^ (pro-aggr., lanes 1, 2) and EC/Tau^ΔK−PP^ mice (anti-aggr., lanes 3, 4) injected with AAV/Tau^ΔK−PP^ presented higher levels of hTau compared to the PBS-injected EC/Tau^ΔK^ mice (lane 5), illustrating that the Tau levels expressed internally and added by AAV injection are additive. **D** Quantification of hTau and mTau shows that EC/Tau^ΔK^ and EC/Tau^ΔK−PP^ (tTA-EC) mice without AAV injection express ~20% of hTau compared to total Tau (also see Fig. 1C). Transgenic mice additionally injected with AAV/Tau^ΔK^ or AAV/Tau^ΔK−PP^ (tTA-EC + AAV) express more hTau (~65% of endogenous mTau; *p* < 0.05). In WT and TKO mice, AAV-mediated hTau expression was ~40% of mTau (*p* < 0.0001). Thus, the ratios of total Tau in the EC of WT mice, transgenic mice, and transgenic mice with AAV injection have ratios of approx. 1:1.2:1.6. Data shown as mean ± SEM

To test whether Tau might spread from the EC to downstream neurons at higher levels and whether spreading might depend on Tau’s competence for aggregation, we increased the expression levels of hTau in EC/Tau^ΔK^ and EC/Tau^ΔK−PP^ mice by unilateral stereotaxic EC injections of AAVs encoding the respective hTau variants (Fig. 4A, B; Suppl. Fig S3). EC/Tau^ΔK^ mice were injected with AAV/Tau^ΔK^, whereas EC/Tau^ΔK−PP^ mice received AAV/Tau^ΔK−PP^ injections. The contralateral control hemispheres received an injection of PBS.

As anticipated, AAV injections increased the level of hTau expression in the injected hemispheres of EC/Tau^ΔK^ and EC/Tau^ΔK−PP^ mice compared to the PBS-injected control hemisphere, whereby the levels of hTau in AAV-injected ECs increased ~ threefold to ~65% of endogenous mTau (Fig. 4C, D). We also injected WT and Tau knockout (TKO) mice with AAV/Tau^ΔK^ and AAV/Tau^ΔK−PP^, which produced hTau levels of ~40% of endogenous mTau in wild-type mice (Fig. 4D). Thus, the ratios of mTau to total Tau in the EC of WT mice, transgenic mice (low expression of Tau^ΔK^ or Tau^ΔK−PP^), and transgenic mice with AAV injections (higher expression of Tau^ΔK^ or Tau^ΔK−PP^) had ratios of approximately (1:1.2:1.6).

### Tau Protein Spreads Between Neurons Independently of Its Aggregation Propensity

Next, we investigated whether the higher levels of hTau in the EC of AAV-injected mice would result in increased spreading of Tau protein and/or Tau pathology. To this end, we visualized AAV transduced cells in the EC utilizing the GFP encoded by the AAV eGFP.P2a.hTauΔK280(-PP) constructs as a separate protein. In analogy with earlier studies (Wegmann et al. 2015, 2019), cells initially transduced by AAV particles were positive for both GFP and hTau (GFP^+^/Tau^+^) and named “donor cells,” as they express hTau that might be transferred to other cells. Cells that had Tau (detected by immunolabeling for human Tau) but not GFP likely received hTau from the initial donor cells via cell-to-cell Tau transfer and were named Tau “recipient cells” (Tau^+^/GFP^─^).

**Fig. 5:**
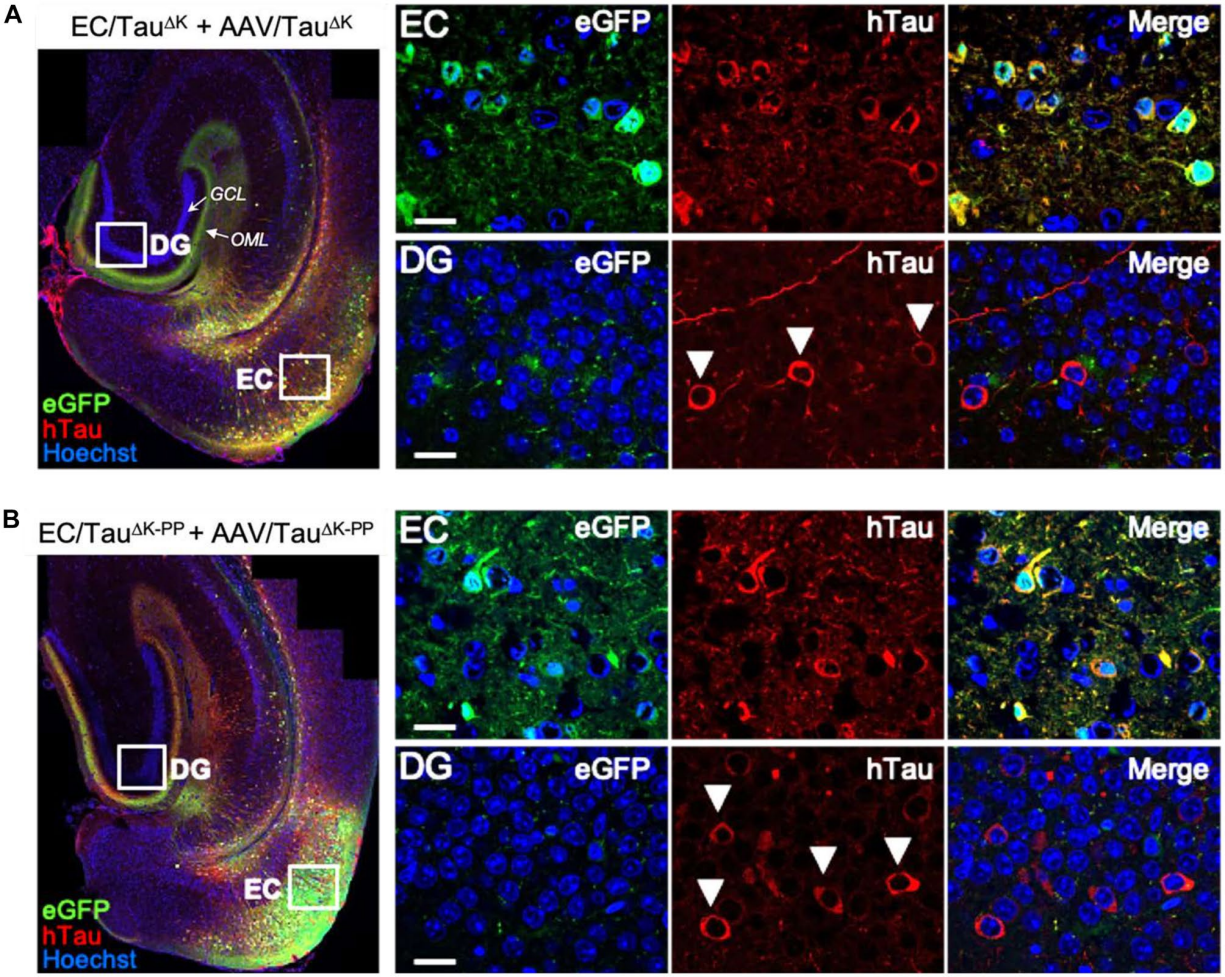
Trans-synaptic spreading of hTau in EC/Tau^ΔK^ and EC/Tau^ΔK−PP^ mice with additional injection of AAV/Tau^ΔK^ or AAV/Tau^ΔK−PP^. **A** Immunolabeling of brain sections with the hTau-specific antibody TauY9 (red) shows hTau (Tau^ΔK^) donor cells in the EC (GFP + /hTau + ; green/red) and hTau recipient cells (GFP-/hTau + ; red) in the DG of AAV/Tau^ΔK^ injected EC/Tau^ΔK^ mice at 3 months p.i.. Axon terminals in the OML are filled with GFP expressed in EC donor cells. The GCL of the DG is visible as a layer of neuronal nuclei (blue). **B** hTau immunolabeling of brain sections shows anti-aggregant hTau (Tau^ΔK−PP^) donor cells in the EC and hTau recipient cells (GFP-/hTau +) in the DG of AAV/Tau^ΔK−PP^ injected EC/Tau.^ΔK−PP^ mice at 3 months p.i. Arrowheads indicate tau recipient in the GCL of the DG of both pro- and anti-aggregant mice. These cells received hTau from Tau donor neurons located in the EC. Scale bar: 100 μm (overview images); 20 μm (higher magnification images)

Three months after AAV injection, both EC/Tau^ΔK^ and EC/Tau^ΔK−PP^ mice showed a low level of hTau recipient neurons in synaptically connected brain areas, the granule cell layer of the DG, and less frequently in the hippocampal areas CA1 and CA3 (Fig. 5A, B; white arrowheads). In WT and TKO mice injected with AAVs encoding Tau^ΔK^ or Tau^ΔK−PP^ (Suppl. Fig. S4), we also detected Tau^+^/GFP^─^ recipient cells outside the EC at comparable levels.

Together these data indicate that hTau protein can be transferred to neurons downstream of the perforant path, which was independent of the Tau aggregation potential and did not rely on the presence of endogenous mTau. There was no sign of pathological changes in the recipient cells.

### Tau Phosphorylation Is More Pronounced in Mice Expressing Pro-aggregant Tau

**Fig. 6:**
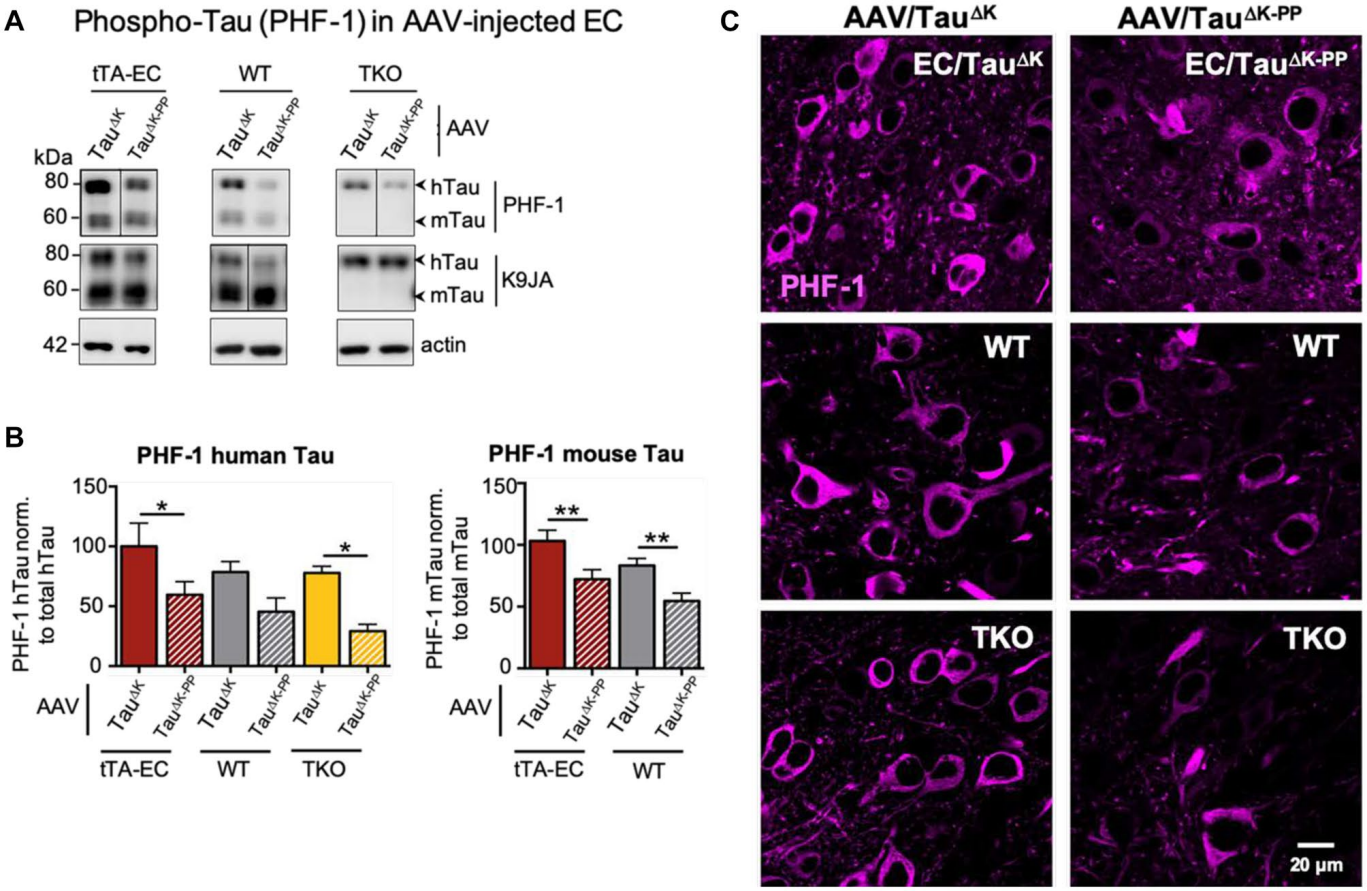
Increased Tau phosphorylation (pS396/pS404) in the EC of AAV/Tau^K^ injected mice. **A** Representative western blot analysis of Tau phosphoprylated at residues Ser396/Ser404 (PHF-1) and of total Tau (K9JA) in EC lysates from tTA-EC mice (EC/Tau^ΔK^ or EC/Tau^ΔK−PP^, lanes 1, 2), WT (lanes 3, 4), and TKO mice (lanes 5, 6), each injected with either AAV/Tau^ΔK^ (lanes 1, 3, 5) or AAV/Tau^ΔK−PP^ (lanes 2, 4, 6) at 3 months p.i. Lanes 1, 2: [tTA-EC + AAV]-induced expression of pro- and anti-aggregant human Tau yields pronounced bands of mTau and hTau, both phosphorylated at the PHF-1 epitope. Lanes 3, 4: [WT + AAV] mice showed pronounced mTau, lesser hTau, but pronounced PHF-1 reactivity only in pro-aggregant hTau. Lanes 5, 6: [TKO + AAV] mice showed only hTau and pronounced PHF-1 reactivity only in the pro-aggregant mice (lane 5, top). The data illustrate that pro-aggregant hTau is more prone to phosphorylation by PHF-1. **B** Differential quantification of phosphorylated human and mouse Tau shows that AAV/Tau^ΔK^ injected mice have generally higher (~30–40%) PHF-1 Tau levels (bars 1, 3, 5) than AAV/Tau^ΔK−PP^ injected animals (bars 2, 4, 6). One-way ANOVA with post-hoc uncorrected Fisher’s LSD test for multiple comparisons. *p < 0.05; **p < 0.01. Data shown as mean ± SEM. **C** Immunolabeling with PHF-1 antibody detects higher amounts of phospho-Tau (pS396/pS404) in brain sections from tTA-EC, WT, and TKO mice injected with AAV/Tau^ΔK^ compared to AAV/Tau^ΔK−PP^ injected animals. Scale bar: 20 µm

We next analyzed the phosphorylation of Tau at the site of the diagnostic antibody PHF-1 (epitope pS396 + pS404), in all experimental groups of AAV-injected mice. Western blot analysis of EC lysates revealed that EC/Tau^ΔK^ mice, compared to EC/Tau^ΔK−PP^ mice and had ~40% more Tau phosphorylation on the PHF-1 epitope (Fig. 6A, B). The levels of phosphorylation at the PHF-1 epitope was also higher in WT (+ 25%) and TKO (+ 45%) mice injected with AAV-Tau^ΔK^ compared to AAV-Tau^ΔK−PP^. Immunolabeling of brain sections with the PHF-1 antibody (Fig. 6C) confirmed these results. PHF-1 positive cells were more prominent in all Tau^ΔK^-expressing mice (EC/Tau^ΔK^ + AAV/Tau^ΔK^; WT + AAV/Tau^ΔK^; TKO + AAV/Tau^ΔK^) compared to Tau^ΔK−PP^-expressing mice (EC/Tau^ΔK−PP^ + AAV/Tau^ΔK−PP^, WT + AAV/Tau^ΔK−PP^; TKO + AAV/Tau^ΔK−PP^). Thus, the presence of pro-aggregant Tau increased the level of Tau phosphorylation compared to anti-aggregant Tau, regardless of the presence of endogenous mTau.

### Pathological Conformation of Tau in Pro-aggregant Mice Remains Restricted to the EC

**Fig. 7:**
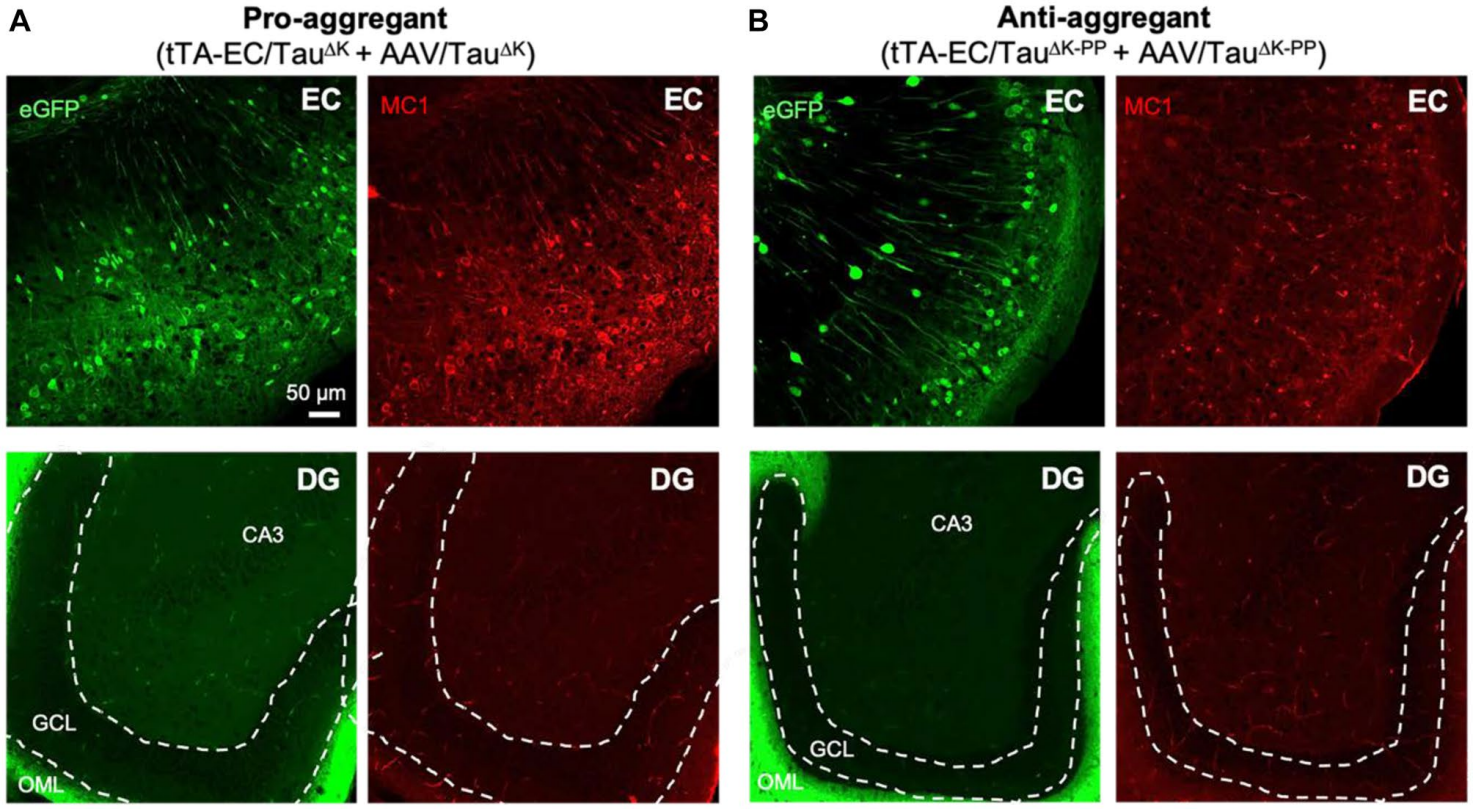
Tau with pathological conformation (MC1 antibody) in AAV-injected EC/Tau^ΔK^ mice. **A** Immunolabeling of misfolded Tau (MC1 positive, red) in brain sections of pro-aggregant EC/Tau^ΔK^ injected with AAV/Tau^ΔK^ showed that a high level of Tau in a pathological conformation was present in the EC and in perforant path axons towards the hippocampus (upper panels), but not further than the outer molecular layer of the DG, where the axon terminals from the EC are located (green band on lower right panel). Therefore, in the pro-aggregant mice no trans-synaptic spreading of pathologic conformation was observed. Images shown are taken 18 months p.i. The granule cell layer (gcl) of the DG is outlined by dashed white lines; ml = molecular layer. Scale bar: 50 μm. **B** Immunolabeling of misfolded Tau (MC1) in brain sections of anti-aggregant EC/Tau^ΔK−PP^ injected with AAV/Tau^ΔK−PP^ shows no Tau with pathological conformation at 18 months p.i

To examine if the increased expression of Tau^ΔK^ and Tau^ΔK−PP^ would also increase the amount of misfolded Tau, we immunolabeled brain sections of AAV-injected EC/Tau^ΔK^ and EC/Tau^ΔK−PP^ mice with the MC1 antibody. Similar to non-injected mice (Suppl. Fig. S2), pathological conformation of Tau (MC1 signal) was only observed in AAV-injected mice expressing Tau^ΔK^ (Fig. 7A), but not in AAV-injected mice Tau^ΔK−PP^ (Fig. 7B). WT and TKO mice injected with AAVs encoding Tau^ΔK^ and Tau^ΔK−PP^ delivered comparable results (data not shown). Importantly, as observed for uninjected EC/Tau^ΔK^ mice, MC1-positve Tau in Tau^ΔK^ expressing mice remained restricted to EC neurons (cell bodies and projections of the perforant pathway) (Fig. 7A). The lack of MC1-positive cells in the DG and hippocampal regions, despite the presence of hTau recipient neurons in these regions, confirmed that markers of Tau pathology did not propagate to downstream neurons, even at enhanced hTau expression after AAV injection, up to 18 months post-injection (p.i.). We conclude that Tau spread can occur without the pre-requisite for its pathological conformation.

### Astrogliosis in Pro-aggregant Tau Mice

**Fig. 8:**
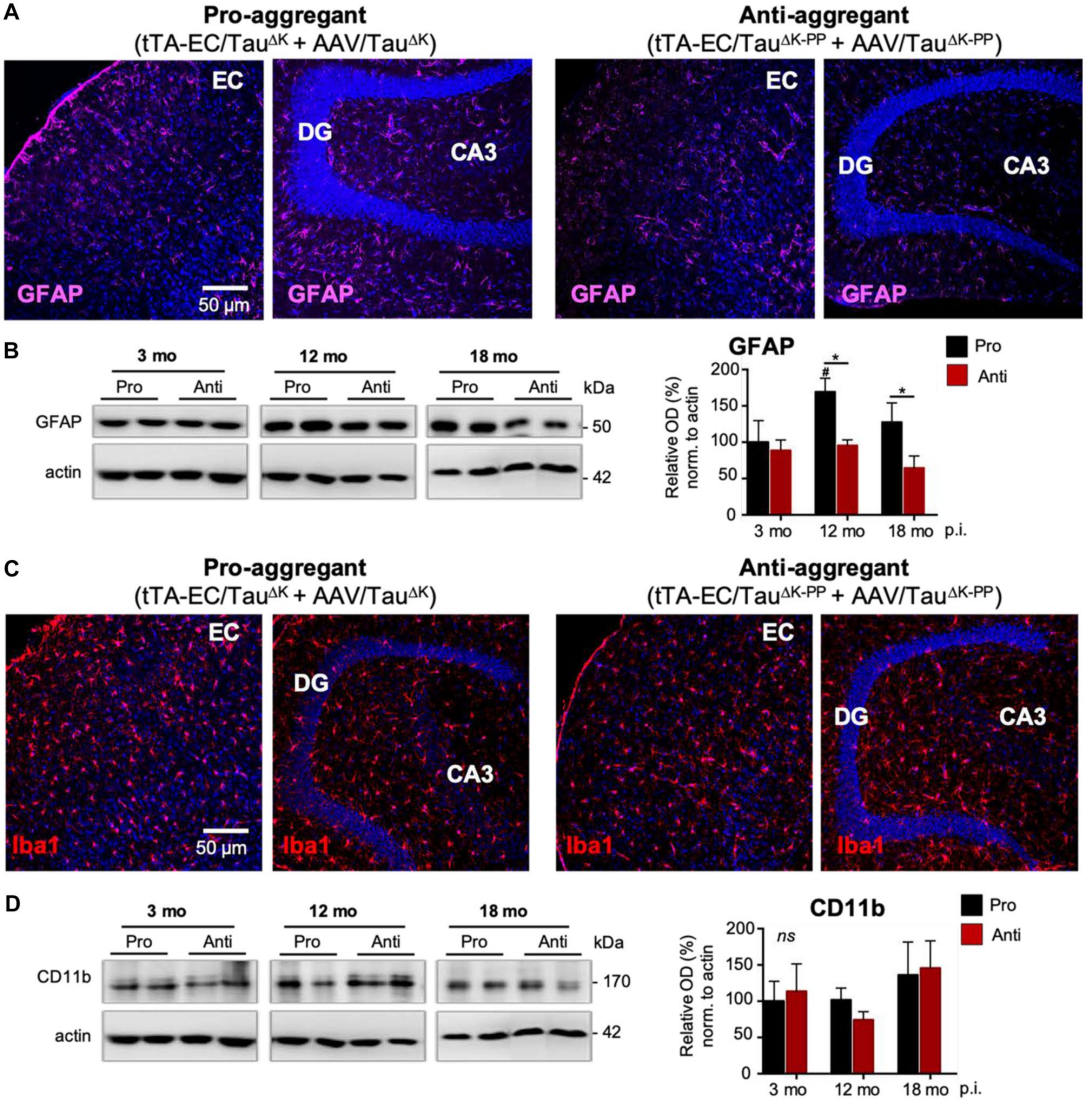
Density of astrocytes and microglia in pro-aggregant and anti-aggregant neuropsin mice. **A** Immunolabeling of astrocytes (GFAP) in brain sections suggests an increased number and fluorescent intensity of astrocytes, reminiscent of a mild astrogliosis, in the hippocampal formation (CA3 and around the DG granule cell layer) of pro-aggregant AAV/Tau^ΔK^-injected tTA-EC/Tau^ΔK^ mice compared to anti-aggregant AAV/Tau^ΔK−PP^-injected tTA-EC/Tau^ΔK−PP^ mice. Scale bar: 50 μm. **B** Western blot analysis of EC lysates shows that GFAP increased in the pro-aggregant mice from 3 to 12 months p.i. (*p* = 0.0227) and was significantly higher (~40%) in AAV/Tau^ΔK^-injected tTA-EC/Tau^ΔK^ mice compared to AAV/Tau^ΔK−PP^-injected tTA-EC/Tau^ΔK−PP^ mice at longer post-injection times of 12 (*p* = 0.0157) and 18 (*p* = 0.0373) months. Two-way ANOVA; with post-hoc uncorrected Fisher’s LSD test. Data shown as mean ± SEM. **C** No differences were observed in the expression of Iba1 between the EC and hippocampal region in the pro-aggregant mice. Regarding Iba1 expression, no differences were observed between pro- and anti-aggregant mice, as well as between the EC and hippocampal region. **D** WB quantification of the levels of the microglial marker CD11b confirmed the absence of differences between pro- and anti-aggregant mice in terms of microglia at the time points analyzed (3, 12 and 18 months p.i.). Data shown as mean ± SEM

Considering a role of microglia and astrocytes in neurodegeneration, and the suggested involvement of microglia in the spreading of Tau pathology (Asai et al. 2015; Ising et al. 2019), we analyzed the expression of glia cell markers as a proxy for gliosis in AAV injected EC/Tau^ΔK^ and EC/Tau^ΔK^ mice. Immunolabeling of brain sections showed higher levels of astrocytic GFAP fluorescence in the hippocampus of mice expressing Tau^ΔK^ compared Tau^ΔK−PP^ from 12 months of age (Fig. 8A). Quantification of GFAP in EC lysates revealed ~70% more GFAP in EC/Tau^ΔK^ compared to EC/Tau^ΔK−PP^ mice at 12 and 18 months p.i. (Fig. 8B). No differences were observed in the number of hippocampal microglia (Iba-positive cells) between EC/Tau^ΔK^ and EC/Tau^ΔK−PP^ mice (Fig. 8C), which was supported western blot quantification of the microglial marker CD11b in EC lysates (Fig. 8D). These data suggest that the expression of pro-aggregant hTau not only increases Tau phosphorylation and misfolding, but also induces astrocyte reactivity in the brain.

### Loss of Synaptic Marker and Body Weight in Pro-aggregant Mice

**Fig. 9:**
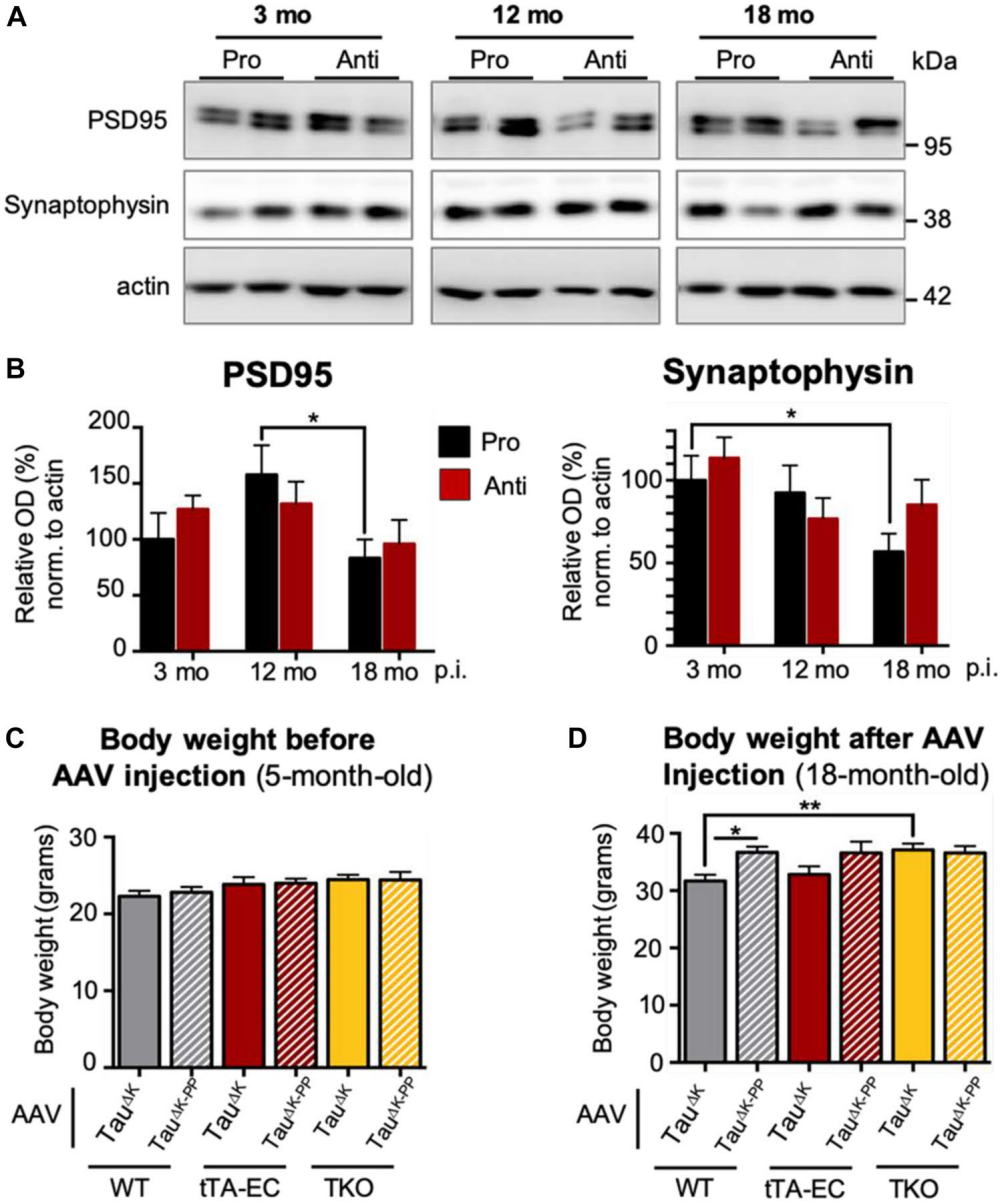
Decrease of synaptic marker synaptophysin and body weight in AAV/Tau^ΔK^ injected tTA-EC/Tau^ΔK^ and WT mice. **A**, **B** Western blot analysis of EC fractions shows a the levels of pre-synaptic synaptophysin decreased by ~40% over time in AAV/Tau^ΔK^ injected tTA-EC/Tau^ΔK^ mice and were significantly lower at 18 compared to 3 months p.i. (*p* = 0.0329). tTA-EC/Tau^ΔK−PP^ mice showed no significant decrease of synaptophysin over time. Data shown as mean ± SEM. Data shown as mean ± SEM. Two-way ANOVA with post hoc analysis with uncorrected Fisher’s LSD test. * denotes the effect of time. *p < 0.05. **C** The body weight (BW) of mice before AAV injection, at 5 months of age, was similar between tTA-EC, WT, and TKO mice later injected with AAV/Tau^ΔK^ or AAV/Tau^ΔK−PP^. **D** At 18 months after AAV injection, WT mice injected with AAV/Tau^ΔK^ had a lower body weight (− 12% BW; *p* = 0.0157) than WT mice injected with AAV/Tau^ΔK−PP^, suggesting a potential detrimental effect of pro-aggregant hTau^ΔK^. A similar tendency was observed in tTA-EC mice injected with AAV/Tau^ΔK^ compared to AAV/Tau^ΔK−PP^ (− 5% BW, ns). In TKO mice, Injection of AAV/Tau^ΔK^ or AAV/Tau.^ΔK−PP^ did not affect the body weight. Data shown as mean ± SEM. One-way ANOVA with uncorrected Fisher’s LSD test for multiple comparisons. *p < 0.05; **p < 0.01

Pathological Tau phosphorylation and misfolding, as well as glia cell activation, is associated with synaptic pathology prior to neurodegeneration in Tau transgenic mice and AD (Spires-Jones et al. 2011) and in AD (Heneka et al. 2018). To investigate the effect of pro- and anti-aggregant Tau on synaptic proteins, we analyzed the levels of pre-synaptic synaptophysin in EC lysates of AAV-injected EC/Tau^ΔK^ and EC/Tau^ΔK−PP^ mice (Fig. 9A, B). Both synaptic markers decreased with age (3 to 18 months), but the effect was more pronounced in EC/Tau^ΔK^ than in EC/Tau^ΔK−PP^ mice.

To obtain insights on the overall animal’s welfare in response to Tau^ΔK^ and Tau^ΔK−PP^ expression, we monitored the body weight (BW) of EC/Tau^ΔK^ and EC/Tau^ΔK−PP^ mice before and after AAV injections. Before AAV injection, at 5 months of age, the BW was similar in EC/Tau^ΔK^ and EC/Tau^ΔK−PP^ compared to WT and TKO mice (Fig. 9C). However, at 18 months p.i., pro-agregant EC/Tau^ΔK^ or WT animals injected with AAV-Tau^ΔK^ had a significant lower BW (− 5%) than their counterparts injected with AAV-Tau^ΔK−PP^ (Fig. 9D). This suggests that elevated levels of Tau^ΔK^ play a detrimental effect in the mice.

## Discussion

**Fig. 10:**
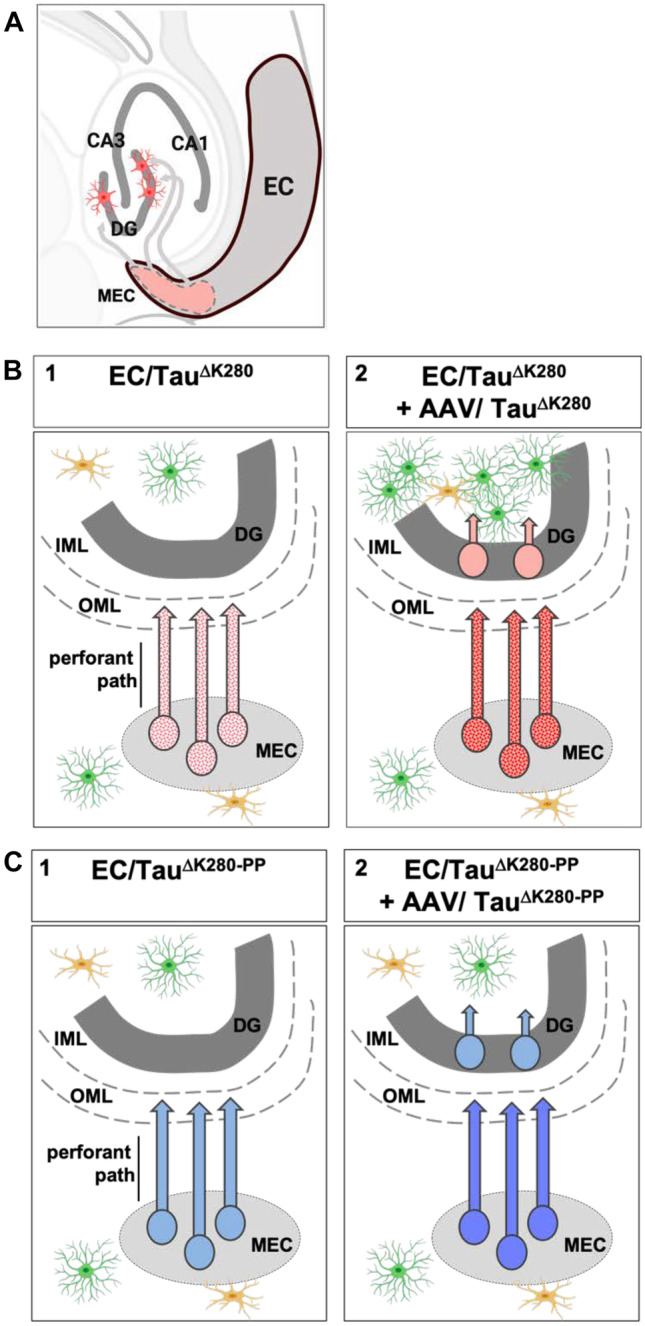
Summary of experimental design and results. **A** Overview of entorhinal cortex and hippocampus. Axons emanating from MEC neurons traverse the perforant path towards the OML where they connect with dendrites emerging from DG neurons; this corresponds to Braak stage 1 of AD. Transgenic human Tau (pink) is expressed only in the MEC neurons and travels by slow anterograde axonal transport to axon terminals where most of it is degraded. Some of Tau may be released and internalized by DG dendrites where it may appear in cell bodies by retrograde transport. **B1** Expression of human pro-aggregant EC/Tau^ΔK^ (stippled red) at low levels leads to hTau-containing axons with incipient hallmarks of misfolding, but no transfer to DG neurons. **B2** Boosting the expression to higher levels via AAV injection increases Tau, misfolding, and some transfer of Tau to DG neurons. In addition, hippocampal astrocytes become activated (green cells). **C1** Expression of human anti-aggregant EC/Tau^ΔK−PP^ (plain blue) at low levels leads to similar Tau transport to axon terminals, but no signs of abnormal changes, and no transfer to DG neurons. **C2** Additional AAV transfection leads to higher level of Tau, with some transfer of Tau to DG neurons, but without abnormal changes, and no activation of astrocytes. The results show that Tau spreads from the EC at similar rates and abnormal changes within neurons are linked to Tau’s aggregation propensity, as well as to activation of astrocytes by an unknown signaling mechanism

To study the effect of Tau aggregation propensity on trans-neuronal Tau protein spread and the propagation of Tau pathology, we used transgenic mice expressing low amounts of full-length human Tau in a pro- or anti-aggregant form (EC/Tau^ΔK280^ or EC/Tau^ΔK280−PP^, respectively) under control of the neuropsin promoter, which restricts expression to the EC (Mayford et al. 2002). The mutants Tau^ΔK^ and Tau^ΔK−PP^are similar in their microtubule interactions but have opposite aggregation propensities. Consequently, mice expressing pro-aggregant Tau develop cognitive deficits whereas mice with anti-aggregant Tau do not (Anglada-Huguet et al. 2023; Van der Jeugd et al. 2012). Assuming that Tau pathology proceeds by spreading of Tau protein, one would expect that pro-aggregant misfolded Tau spreads more efficiently than anti-aggregant Tau, carrying forward the pathogenic activity. In our studies, we find that both pro- and anti-aggregant human Tau distribute similarly in expressing EC neurons and accumulate in their axon terminals in the OML (Fig. 10A). The transfer of Tau to neurons in the DG occurred only after boosting hTau expression by additional AAV-mediated delivery (from 20 to 65% of endogenous Tau) (compare Fig. 10B1, B2) and was comparable to previous studies (de Calignon et al. 2012; Wegmann et al. 2015). The pathogenicity of pro-aggregant Tau manifested itself not in its spreading potential, but in its pathological change to a misfolded pre-tangle state (MC1) and increased phosphorylation in the EC neurons (Augustinack et al. 2002) (Fig. 10B2). These changes were not transferred to Tau recipient neurons in the DG, where hTau was not misfolded. This indicates that the spreading of hTau is independent of its potential for misfolding (compare Fig. 10B2 vs. C2). Remarkably, the most obvious evidence for pathological changes in pro-aggregant mice was the increase in hippocampal astrocytes (Fig. 10C2), even without misfolding of Tau spreading into the DG. Thus, Tau^ΔK^expressing EC neurons may signal their pathogenic state(s) to other cells, including astrocytes, through ways distinct from the transfer of misfolded Tau. Hippocampal astrogliosis could, for example, be triggered by release of misfolded Tau or other signaling molecules from EC neurons in the extracellular fluid (Yamada et al. 2011). Similarly, hTau^ΔK^recipient neurons in the DG could release factors that trigger astrocyte activation in their vicinity, even before the accumulation of pathological Tau in their cytosol (Walsh and Selkoe 2016).

One caveat in the interpretation is the regional specificity of the neuropsin promoter which has been questioned, arguing that it can be active outside the EC as well (Yetman et al. 2016). In our mice, we did not find hTau positive cells outside of the EC in EC/Tau^ΔK^ and EC/Tau^ΔK−PP^mice up to 24 months of age. The discrepancy between the results might be accounted for by several factors, including the use of different methods to check the specificity of Nop-tTA expression (de Calignon et al. 2012; Liu et al. 2012; Harris et al. 2010; Rowland et al. 2013). Furthermore, other factors may influence the expression pattern of some promoters, including age (Long and Rossi 2009), epigenetic modifications (Swain et al. 1987; Akitake et al. 2011), and the strain background on which the transgene is expressed (Han et al. 2012; Strong et al. 2012).

However, even the very low Tau^ΔK280^ expression in our EC/Tau^ΔK^ mice was sufficient to induce Tau changes in the EC reminiscent of early pathological alterations in AD: we observed phosphorylated Tau (pS262/pS356) and pathological conformation (MC1 antibody) in the EC of pro-aggregant Tau^ΔK^ mice as early as 3 months. Phosphorylation and pathological conformation of Tau in EC/Tau^ΔK^ were absent outside the EC, indicating that no pathological Tau was propagated. Furthermore, EC/Tau^ΔK^ animals did not show Gallyas- or ThioS-positive neurons in the EC. We attribute these differences to previous studies in similar mouse models mostly to the very low amount of human Tau expression in our mouse models compared to the other models.

Because we suspected that the absence of trans-synaptic hTau spreading in our EC/Tau^ΔK^ and EC/Tau^ΔK−PP^ mice was due to the low transgene expression, we combined these mouse models with stereotaxic EC-injections of AAVs encoding the same hTau versions (Tau^ΔK^ and Tau^ΔK−PP^) already genetically expressed in the mice. This led to an ~45% increase in hTau expression, equal to a 0.65-fold overexpression of hTau over mTau, which one may still consider a mild physiological overexpression. When increasing the levels of hTau expression in the EC by AAV-injections, Tau recipient neurons (hTau + /GFP─) occurred in regions with direct anatomical connections to the EC, namely in the granule cell layer of the DG and the hippocampal regions CA1 and CA3. In previous studies using AAV-mediated hTau expression in the EC, we observed hTau spreading also to other more distant connected regions in the brain, such as the contralateral hippocampus and the olfactory cortex (Wegmann et al. 2019); we did not assess these regions in this study. Interestingly, and against our initial hypothesis, the hTau spread appeared to be similar for both pro- and anti-aggregant hTau. We did not detect further spreading of hTau to the following cell layers or into the contralateral hemisphere. Remarkably, we never detected MC1-positive misfolded Tau outside of EC neurons, showing that the ability of Tau protein to spread from cell to cell was independent of the protein’s aggregation potential and its pathological conformation (revealed by MC1 antibody), as it was postulated for the case of PrPSc (Prusiner 2012).

Furthermore, both Tau^ΔK^ and Tau^ΔK−PP^could spread across cells even in the absence of endogenous mouse Tau, supporting the idea that templated misfolding is not necessary for Tau spreading (Wegmann et al. 2015). These features argue for a model, in which the appearance of Tau in neighboring cells is due to physiological trans-synaptic spreading of non-pathological Tau, which depends on the concentration but not pathological conformation of Tau in the donor cell. Release and uptake of Tau may thus be part of a continuous exchange that is not necessarily part of a pathological process.

Previous studies showed that stereotactic injection of pre-aggregated Tau”seeds” into mutant Tau transgenic mice leads to an amplification of Tau aggregation due to seeded aggregation of the overexpressed pro-aggregant Tau (Clavaguera et al. 2009); injection of pre-formed Tau fibrils into wild-type mice produces no effect in non-transgenic mice (Peeraer et al. 2015). Despite the relevance of these approaches as in vivo models for templated Tau aggregation, they do not reflect the situation in sporadic AD patients, where non-mutant Tau aggregates first in the EC and from there progresses to other brain regions. In our study, and related approaches (de Calignon et al. 2012; Liu et al. 2012; Wegmann et al. 2019; Amaral et al. 2021), the accumulation of hTau is restricted to the EC, which mimics early stages of Tau pathology in AD.

Our data from AAV-injected EC/Tau^ΔK^ mice show that phosphorylated Tau and Tau in a pathological conformation (MC1) was confined to neurons with Tau^ΔK^ expression in the EC, but did not and occur in other brain regions. Only pro-aggregant hTau expressing neurons were filled with MC1-positive up to their axon terminals in the outer molecular layer of the DG, i.e., consistent with the intracellular transport of Tau. These results are consistent with previous findings for AAV-mediated expression of Tau^P301L^in the EC (Wegmann et al. 2019). Since Tau is transported by slow axonal transport (~0.2–0.4 mm/day) (Mercken et al. 1995), the distance from neuronal somata in the EC to the axon terminal in the OML (a few millimeters in mouse brain) would be traversed in ~3–5 weeks, much faster than the observed onset of pathological changes in EC/Tau^ΔK^mice (at ~12 months). Possibilities of how Tau may be released from and transferred between neurons include the exchange of cytoplasmic content via exosomes and other vesicles (Yamada et al. 2011; Dujardin et al. 2014) which was suggested to contribute to the spreading of Tau protein together with other proteins and RNA (Wang et al. 2017; Gamez-Valero et al. 2019). Cellular senescence in the brain (Musi et al. 2018) and neuronal activity (Yamada et al. 2014; Wu et al. 2016) seems to also contribute to the release of Tau to the extracellular space and its propagation to other cells in the brain.

Recent evidence suggested that neuroinflammation may be the culprit for Tau pathology observed in AD (Ising et al. 2019; Vogels et al. 2019; Heneka et al. 2018; Ishizawa et al. 2004; Zhang et al. 2013; Leyns and Holtzman 2017), and both microglia and astrocytes have been suggested to contribute to the onset and propagation of Tau pathology in the brain. Furthermore, microglia have been suggested to directly enable Tau protein spreading via exosome secretion, contributing to the progression of the pathology (Asai et al. 2015; Wang et al. 2017). Based on the evidences implicating microglia in the pathogenesis and spreading of Tau, we expected to observe higher levels of microglia in the pro-aggregant models compared to the anti-aggregant mice; however, analysis of microglia markers in AAV-injected EC/Tau^ΔK^ and EC/Tau^ΔK−PP^ mice revealed no differences.

In Tau transgenic mouse models, astrocyte activation occurs in brain regions with Tau pathology, often before the development of mature plaques and/or tangles, suggesting that astrocytosis may precede the development of tangles or plaques and is involved in AD pathogenesis (Garwood et al. 2010). In AAV-injected EC/Tau^ΔK^ mice, increased expression of GFAP indicated astrogliosis. A similar elevation of GFAP but not Iba1 levels was previously observed for Tau^P301L^compared to WT Tau (Wegmann et al. 2019). Interestingly, the pure presence of Tau^ΔK^ at the axon terminals of the performant path was not sufficient to induce a glia reaction in EC/Tau^ΔK^ mice. Only in conditions of Tau spreading (at mild overexpression in AAV-injected EC/Tau^ΔK^ mice), we detected astrocyte activation, suggesting that extracellular Tau^ΔK^may have directly triggered this reaction and/or that astrocytes may be involved in Tau spreading. Similarly, the presence of extracellular Tau upon direct brain injections of pre-aggregated Tau was suggested to trigger neuroinflammatory signals leading to microglia and astrocyte activation and phagocytosis (Peeraer et al. 2015). In fact, also the time-dependent decrease of synaptophysin in AAV-injected EC/Tau^ΔK^ mice suggested that the presence of misfolded pro-aggregant Tau^ΔK280^ in the EC triggered pathological effects sufficient to inflict synaptic deficits in the brain.

## Conclusions

The trans-synaptic spreading of Tau proteins appears to be independent of their aggregation propensity and does not rely on Tau pathology markers (phosphorylation and misfolding). However, even near physiological amounts of pro-aggregant Tau induce Tau phosphorylation and misfolding in expressing in EC neurons and are associated with synaptic alterations and astrogliosis, reminiscent of early pathological changes in the AD brain. Since glia cell activation may precede and facilitate Tau spreading and toxicity in AD, further studies need to investigate the inflammatory mechanisms in these processes. Furthermore, we propose that it is of major relevance to clearly distinguish between the spreading of Tau protein across cells and the propagation of Tau pathology throughout the brain in AD.

### Supplementary Information

Below is the link to the electronic supplementary material.

Supplementary file1 (PDF 1608 KB)

## Data Availability

All data generated or analyzed during this study are included in this published article [and its supplementary information files].

## Abbreviations

AAV/Tau^ΔK^: Adeno-associated virus encoding Tau^ΔK^
AAV/Tau^ΔK^^−^^PP^: Adeno-associated virus encoding Tau^ΔK^^−^^PP^
AAV: Adeno-associated-virus
anti-aggregant Tau: Tau containing mutations ΔK280, I277P, I308P, = Tau^ΔK^^−^^2P^
AD: Alzheimer disease
Aβ: Amyloid beta
BLI: Bioluminescence imaging
BW: Body weight
CA1: Cornu ammonis 1
CA2: Cornu ammonis 2
CA3: Cornu ammonis 3
CNS: Central nervous system
DG: Dentate gyrus
EC: Entorhinal córtex
Gcl: Granule cell layer
GFAP: Glial fibrillary acidic protein
GFP: Green fluorescent protein
hTau: Human Tau
KO: Knockout
LEC: Lateral entorhinal cortex
MAP: Microtubule-associated protein
Ml: Molecular layer
MEC: Medial entorhinal cortex
MT: Microtubule
NFTs: Neurofibrillary tangles
Nop: Neuropsin
PaS: parasubiculum
PHF: Paired helical filament
p. i.: Post-injection
pro-aggregant Tau: Tau containing mutation ΔK280, = Tau^ΔK^
Tau: Protein encoded by human *MAPT* gene (UniprotKB P10636-8), alias 2N4R-Tau, hTau
ThS: Thioflavin S
TKO: Tau knockout mouse line
tTA: Tetracycline transactivator
tTA-EC/Tau^ΔK^ (for short EC/Tau^ΔK^): Mouse line expressing Tau^ΔK^ in entorhinal cortex under the neuropsin promoter
tTA-EC/Tau^ΔK^^−^^2P^ (for short EC/Tau^ΔK^^−^^2P^): Mouse line expressing Tau^ΔK^^−^^2P^ in entorhinal cortex under the neuropsin promoter
WB: Western blot
WT: Wild-type
