# Supplementary material for: Spreading of Tau Protein Does Not Depend on Aggregation Propensity
Source: J Mol Neurosci. 2023 Aug 22;73(9-10):693–712. doi: 10.1007/s12031-023-02143-w (PMC10694122; doi:10.1007/s12031-023-02143-w)
Supplement: Supplementary file 1 — Supplementary file1 (PDF 1608 KB) [file 12031_2023_2143_MOESM1_ESM.pdf]

SUPPLEMENTAL FIGURE S1

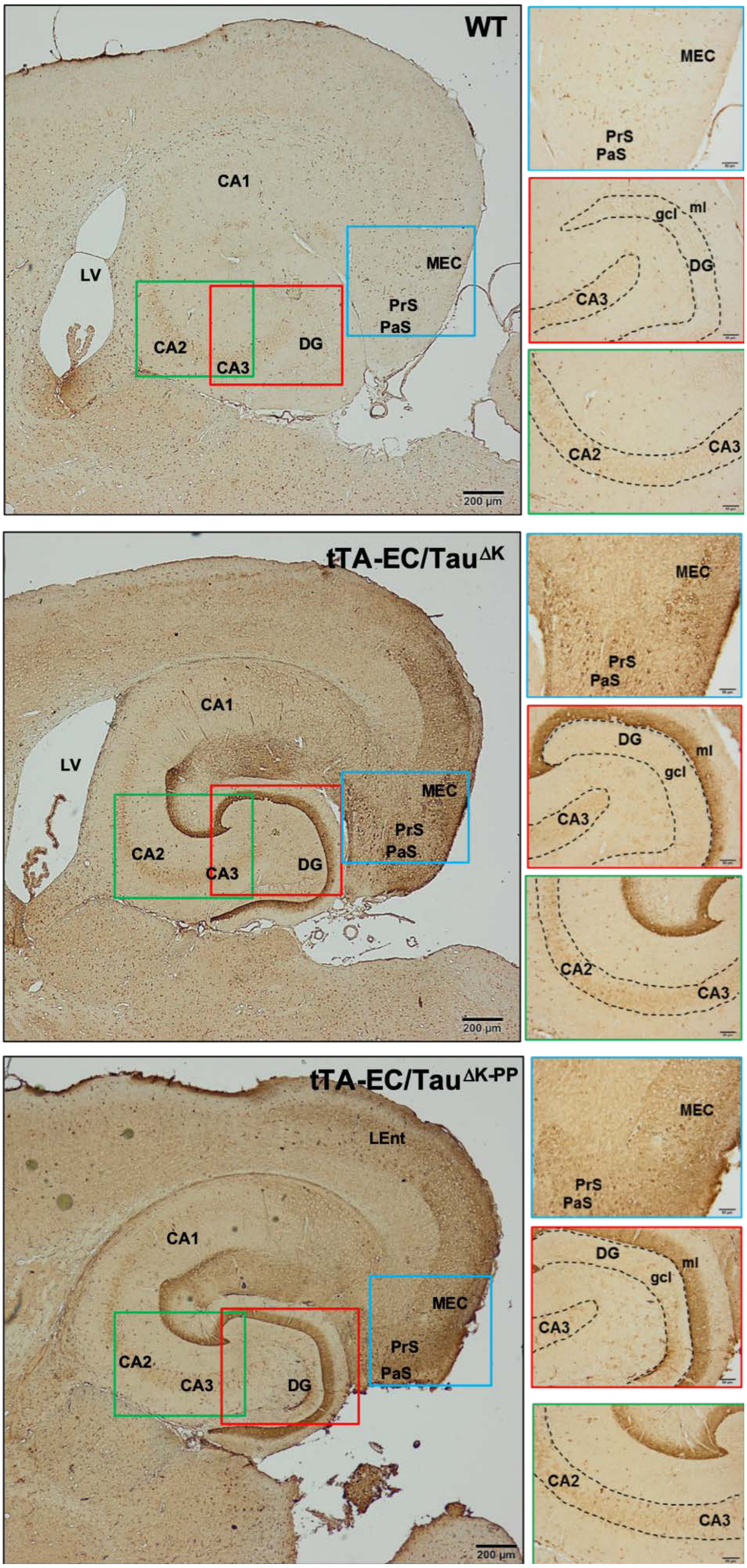

SUPPLEMENTAL FIGURE S2

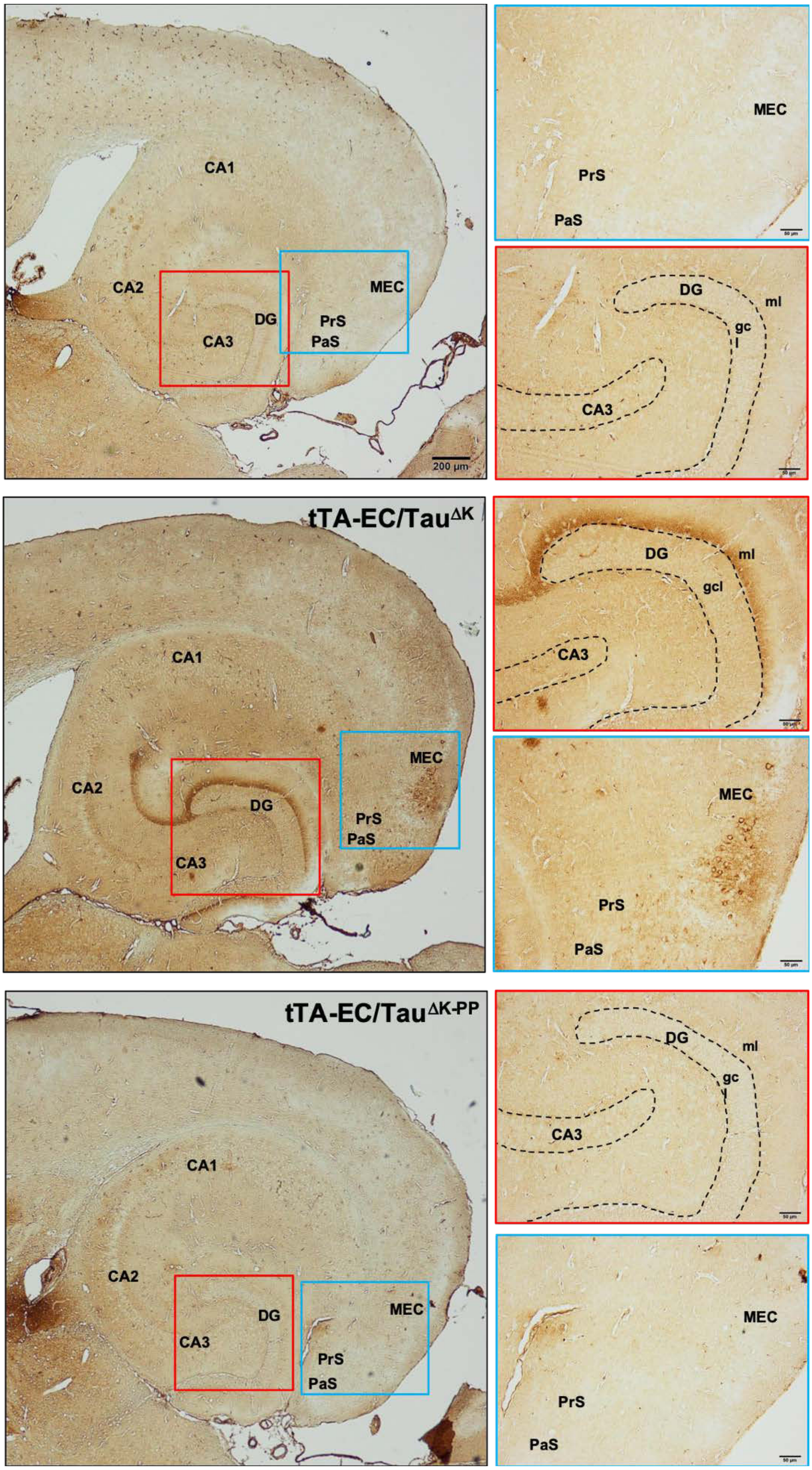

SUPPLEMENTAL FIGURE S3

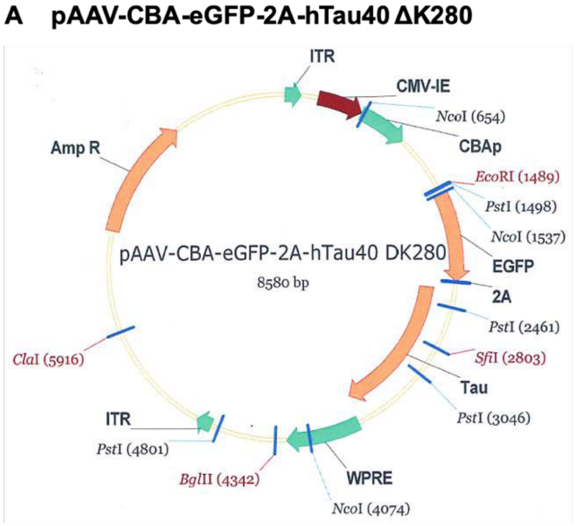

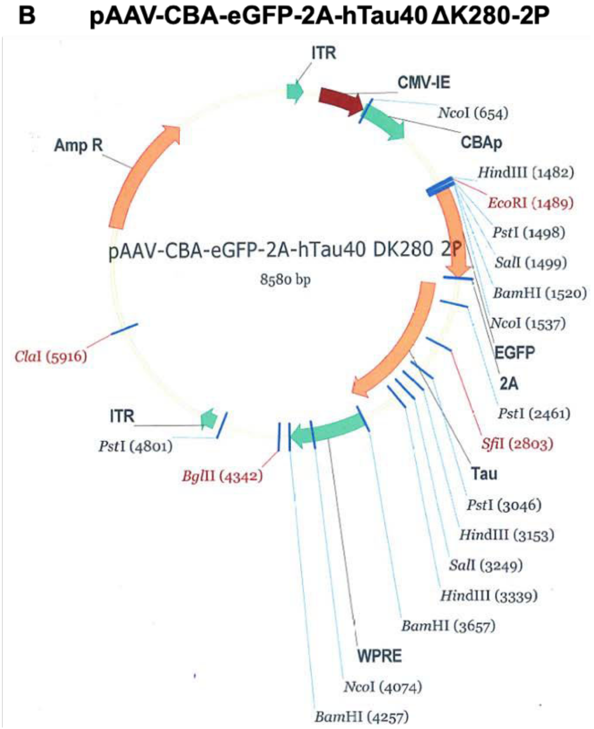

SUPPLEMENTAL FIGURE S4

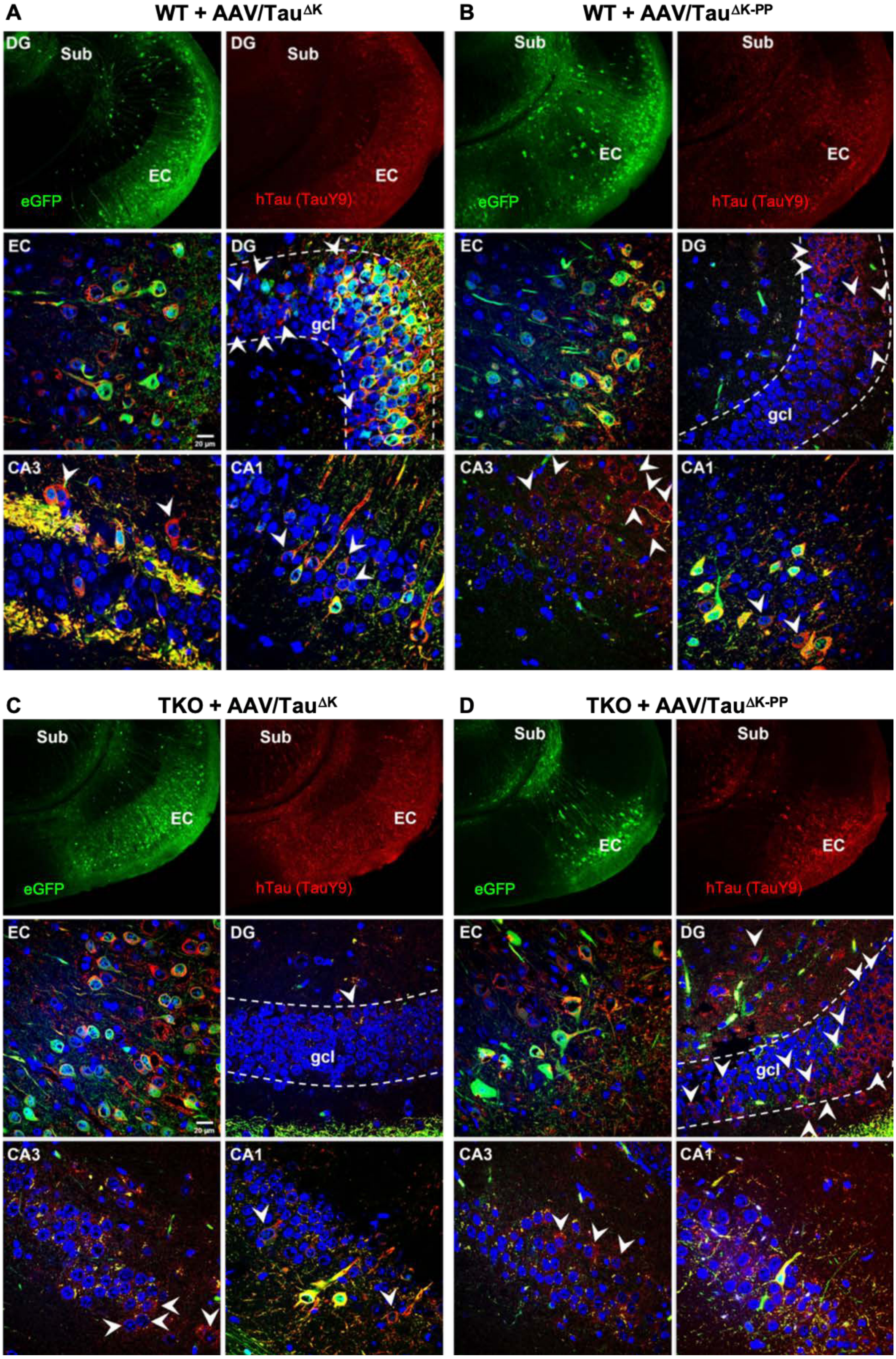

Supplementary figures

SuppFig. S1: Expression of hTau in 24-month-old tTA-EC/Tau$^{\Delta K}$ and tTA-EC/Tau$^{\Delta K\text{-}PP}$ mice.

Immunolabeling ofhTau (human-specific Tau antibody HT7) in horizontal brain sections shows that in both pro-aggregant tTA-EC/Tau$^{\Delta K}$ and anti-aggregant tTA-EC/Tau$^{\Delta K\text{-}PP}$ mice the expression of hTau remains restricted to EC neurons and their axons of the perforant path, up to the molecular layer of the DG. No trans-synaptic spreading of Tau protein to DG granule cells was observed, as we did not detect HT7+ cells in brain regions other than the EC. WT mice (left panel) did not express hTau. Scale bar: 200 µm (overview images); 50 µm (close-up images).

SuppFig. S2: Expression of Tau with pathological conformation in 24-month-old tTA-EC/Tau$^{\Delta K}$ mice.

Immunolabeling of misfolded Tau (conformational Tau antibody MC1) in horizontal brain sections detects Tau with a pathological conformation only in EC neurons of pro-aggregant tTA-EC/Tau$^{\Delta K}$ mice. The occurrence of MC1+ Tau remains restricted to EC neurons and their axons of the perforant path, up to the molecular layer of the DG. These results support observations at earlier time points (e.g. 12-month-old mice, Fig. 3) and in previous studies (23), confirming that Tau pathology, even in aged mice, is restricted to the EC and perforant pathway and does not propagate to other cell layers. Anti-aggregant tTA-EC/Tau$^{\Delta K\text{-}PP}$ and WT mice do not have Tau with pathological conformation. Scale bar: 200 µm (overview images); 50 µm (close-up images).

SuppFig. S3: Plasmid maps of adeno-associated viruses (AAVs) used in the study.

(A) pAAV-CBA-eGFP-2A-hTau40$^{\Delta K280}$, for the expression of pro-aggregant full-length mutant human Tau (2N4R) with the ΔK280 mutation.

(B) pAAV-CBA-eGFP-2A-hTau40$^{\Delta K280\text{-}2P}$ for the expression of anti-aggregant full length mutant human Tau with the ΔK280 mutation and two beta-breaking prolines.

SuppFig. S4: Trans-synaptic spreading of hTau protein in WT and TKO mice injected with AAV/Tau$^{\Delta K}$ or AAV/Tau$^{\Delta K\text{-}PP}$ 3 months p.i.

(A+B) Immunolabeling of hTau (antibody TauY9) in brain sections revealed the presence of hTau positive cells in DG, CA3 and CA1 regions of WT mice injected with pro-aggregant AAV/Tau$^{\Delta K}$ and AAV/Tau$^{\Delta K\text{-}PP}$. hTau recipient cells (GFP-/hTau+) are indicated with arrowheads.

(C+D) Immunolabeling of hTau (antibody TauY9) in brain sections revealed the presence of hTau positive cells in DG, CA3 and CA1 regions of TKO mice injected with pro-aggregant AAV/Tau$^{\Delta K}$ and AAV/Tau$^{\Delta K\text{-}PP}$. hTau recipient cells (GFP-/hTau+) are indicated with arrowheads. Scale bar: 100 µm (overview images); 20 µm (higher magnification images).
